# The glycoprotein 5 of porcine reproductive and respiratory syndrome virus stimulates mitochondrial ROS to facilitate viral replication

**DOI:** 10.1128/mbio.02651-23

**Published:** 2023-12-04

**Authors:** Shuang Zhang, Lei Zeng, Bing-Qian Su, Guo-Yu Yang, Jiang Wang, Sheng-Li Ming, Bei-Bei Chu

**Affiliations:** 1 College of Veterinary Medicine, Henan Agricultural University, Zhengzhou, Henan Province, China; 2 Key Laboratory of Animal Biochemistry and Nutrition, Ministry of Agriculture and Rural Affairs of the People’s Republic of China, Zhengzhou, Henan Province, China; 3 Key Laboratory of Animal Growth and Development, Zhengzhou, Henan Province, China; 4 International Joint Research Center of National Animal Immunology, Henan Agricultural University, Zhengzhou, Henan Province, China; 5 Ministry of Education Key Laboratory for Animal Pathogens and Biosafety, Zhengzhou, Henan Province, China; 6 Longhu Advanced Immunization Laboratory, Zhengzhou, Henan Province, China; Icahn School of Medicine at Mount Sinai, New York, New York, USA

**Keywords:** PRRSV, ER-mitochondria contacts, IP3R, VDAC1, autophagy, NLRP3 inflammasome

## Abstract

Viruses have evolved sophisticated mechanisms to manipulate host cell organelles to serve as niches for persistence and proliferation. In the present study, we aimed to investigate the role of cellular organelles in the replication of porcine reproductive and respiratory syndrome virus (PRRSV). We found that the morphology of mitochondria and the endoplasmic reticulum (ER) were both altered, and the contact between these two organelles was enhanced during PRRSV infection. By the overexpression of PRRSV-encoded open reading frames, we identified that only glycoprotein 5 (GP5) was essential for ER-mitochondria contact. Further investigation revealed that GP5 interacted with the ER inositol 1,4,5-triphosphate receptor (IP3R) and the mitochondrial voltage-dependent anion channel (VDAC1) to promote the Ca^2+^ efflux from ER into mitochondria. Excessive mitochondrial Ca^2+^ uptake resulted in mitochondrial dysfunction and substantial mitochondrial reactive oxygen species (mROS) production. Elevated mROS activated autophagy through the AMPK/mROR/ULK1 axis to facilitate PRRSV replication. GP5-induced mROS also triggered the NOD-like receptor family pyrin domain-containing protein 3 (NLRP3) inflammasome. Inhibition of autophagy augmented NLRP3 inflammasome activation and exhibited an anti-PRRSV effect, suggesting autophagy counteracted the NLRP3-mediated innate immune response. Overall, our findings highlighted the importance of cellular organelles in virus-host interactions and provided new insights into the complex interplay between virus replication and innate immune responses.

**IMPORTANCE:**

Porcine reproductive and respiratory syndrome virus (PRRSV) presents a significant economic concern for the global swine industry due to its connection to serious production losses and increased mortality rates. There is currently no specific treatment for PRRSV. Previously, we had uncovered that PRRSV-activated lipophagy to facilitate viral replication. However, the precise mechanism that PRRSV used to trigger autophagy remained unclear. Here, we found that PRRSV GP5 enhanced mitochondrial Ca^2+^ uptake from ER by promoting ER-mitochondria contact, resulting in mROS release. Elevated mROS induced autophagy, which alleviated NLRP3 inflammasome activation for optimal viral replication. Our study shed light on a novel mechanism revealing how PRRSV exploits mROS to facilitate viral replication.

## INTRODUCTION

In the late 1980s, the porcine reproductive and respiratory syndrome virus (PRRSV) appeared as an unidentified ailment that spread through swine populations in both Europe and North America (1, 2). PRRSV infection is currently one of the most important pig diseases in the world (3, 4). The genome of PRRSV is approximately 15 kb in size, composed of at least 9 overlapping open reading frames (ORFs) (5). The GP5 protein, encoded by ORF5, plays an important role in viral infectivity. It is partnered with the ORF6-encoded matrix protein to allow virus attachment and assembly (6, 7). It has been established that GP5 interacts with salivary adhesins, to allow viral entry into susceptible cells (8). Non-myosin heavy chain 9 has been observed to interact with GP5 and is implicated in the internalization and cell-to-cell spread of PRRSV (9). Moreover, studies have demonstrated that host cathepsin E cleaved GP5, thereby facilitating PRRSV membrane fusion (10). Nonstructural protein 2 (NSP2) is a multifunctional protein that plays a crucial role in the replication and pathogenesis of PRRSV (11, 12). Several studies have shown that the inhibition of NSP2 activity reduced viral replication and attenuated disease symptoms in infected pigs (13). NSP2 has been shown to interact with several host proteins, including the endoplasmic reticulum (ER) chaperone protein BiP (14), which is important for proper protein folding, and the nuclear factor kappa B (NF-kB) (15), which is a key regulator of the immune response.

The ER is a network of membrane-bound tubes and sacs that is involved in protein synthesis, lipid synthesis, and Ca^2+^ storage (16), and mitochondria are organelles that are responsible for generating energy through oxidative phosphorylation (17). ER and mitochondria are physically linked by specialized contact sites called mitochondria-associated membranes (MAMs), which allow the exchange of lipids, Ca^2+^, and other signaling molecules (18). Some viruses have been shown to manipulate the ER-mitochondria interaction to facilitate their replication and survival within host cells. For example, the hepatitis C virus (HCV) induces the formation of convoluted membranes derived from the ER that interact with mitochondria, creating a specialized environment for HCV replication (19). The SARS-CoV-2 virus has also been found to interact with the ER and mitochondria in host cells, possibly contributing to the severe respiratory symptoms observed in infected individuals (20). The human cytomegalovirus has been shown to disrupt the ER-mitochondria interaction to evade the host immune response (21). Overall, the interaction between viruses and ER-mitochondria is complex and multifaceted, and understanding the mechanisms involved could lead to the development of new therapeutic strategies to combat viral infections.

The NOD-like receptor family pyrin domain-containing protein 3 (NLRP3) inflammasome is a multiprotein complex that plays a key role in the innate immune response. It is composed of three main components: NLRP3, apoptosis-associated speck-like protein containing a CARD (ASC), and pro-caspase-1 (22). When cells sense danger signals, such as pathogen-associated molecular patterns or reactive oxygen species (ROS) (23), NLRP3 undergoes conformational changes and oligomerization, leading to the recruitment of ASC and pro-caspase-1. This results in the formation of the NLRP3 inflammasome complex. Once activated, the NLRP3 inflammasome cleaves pro-caspase-1 into its active form, which then promotes the maturation and release of pro-inflammatory cytokines, such as interleukin (IL)-1β and IL-18 (24). These cytokines play crucial roles in initiating and amplifying the inflammatory response, recruiting immune cells, and promoting tissue repair. The NLRP3 inflammasome has been shown to play a role in the immune response against several viruses, such as influenza, herpes, and respiratory syncytial viruses (25–27).

In this study, we revealed a novel mechanism of PRRSV replication, in which the virus manipulated mitochondrial-ER contact through the interaction between PRRSV GP5, IP3R, and VDAC1 to induce excessive Ca^2+^ efflux into mitochondria, resulting in mitochondrial dysfunction and mROS production. The increased mROS activated autophagy to repress the NLRP3 inflammasome and facilitated PRRSV replication.

## RESULTS

### PRRSV infection induces the morphological alterations of mitochondria and ER, and ER-mitochondria contact

**Fig 1:**
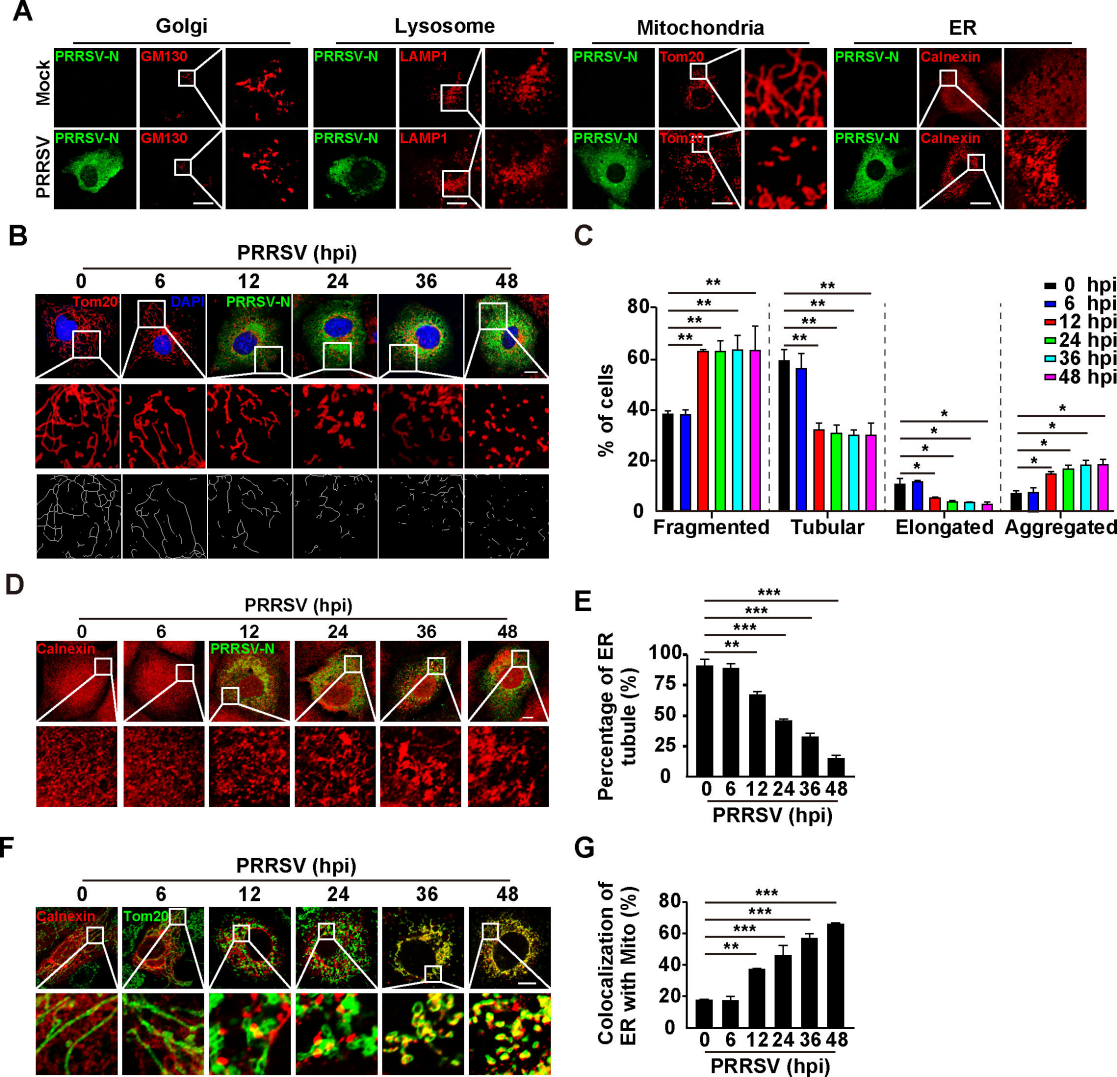
PRRSV infection induces the morphological alterations of mitochondria and ER, and ER-mitochondria contacts. (**A**) MARC-145 cells were mock-infected or infected with PRRSV (MOI = 1) for 24 h. The morphologies of Golgi (GM130), lysosomes (LAMP1), mitochondria (Tom20), and ER (calnexin) were monitored by immunofluorescence analysis. Scale bar: 10 µM. (**B**) MARC-145 cells were infected with PRRSV (MOI = 1) for 0–48 h. The morphology of mitochondria (Tom20) was monitored by immunofluorescence analysis. Scale bar: 10 µM. (**C**) Quantification of the fragmented, tubular, elongated, and aggregated mitochondria from (**B**) (*n* = 30). ^*^P < 0.05, ^**^P < 0.01. (**D**) MARC-145 cells were infected with PRRSV (MOI = 1) for 0–48 h. The morphology of ER (calnexin) was monitored by immunofluorescence analysis. Scale bar: 10 µm. (**E**) Quantification of the percentage of ER tubule from (**D**) (*n* = 30). ^**^P < 0.01, ^***^P < 0.001. (**F**) MARC-145 cells were infected with PRRSV (MOI = 1) for 0–48 h. The co-localization of ER (calnexin) with mitochondria (Tom20) was monitored by immunofluorescence analysis. Scale bar: 10 µm. (**G**) Quantification of the co-localization of ER with mitochondria from (**F**) (*n* = 30). ^**^P < 0.01, ^***^P < 0.001.

To examine the role of cellular organelles in the replication of PRRSV, we first examined the morphological alterations in different organelles after PRRSV infection. Immunofluorescence analysis was performed with specific antibodies against GM130 (Golgi), LAMP1 (lysosomes), Tom20 (mitochondria), and calnexin (ER). Obvious morphological alterations were observed in the mitochondria and ER at 24 h after PRRSV infection (Fig. 1A). There was no significant difference in lysosomal and Golgi morphology before and after PRRSV infection (Fig. 1A). We then infected MARC-145 cells with PRRSV at different time points and quantified mitochondrial morphology alteration. As shown in Fig. 1B and C, the mitochondria became fragmented and aggregated at 12 h post-infection (hpi), and tubular and elongated mitochondria declined significantly. Condensed ER was observed with increases in the length of PRRSV infection, resulting in a decreased percentage of ER tubules in the cells (Fig. 1D and E). These results suggested that the morphologies of mitochondria and ER were simultaneously altered by PRRSV infection. To examine whether these two organelles were correlated, we analyzed their interactions. Immunofluorescence analysis indicated that the fluorescent signals of calnexin and Tom20 were overlaid, suggesting that PRRSV infection induced ER-mitochondria contact (Fig. 1F and G). Taken together, our data indicated that PRRSV infection altered the morphologies of mitochondria and ER to induce their contact.

### PRRSV GP5 stimulates ER-mitochondria contact

**Fig 2:**
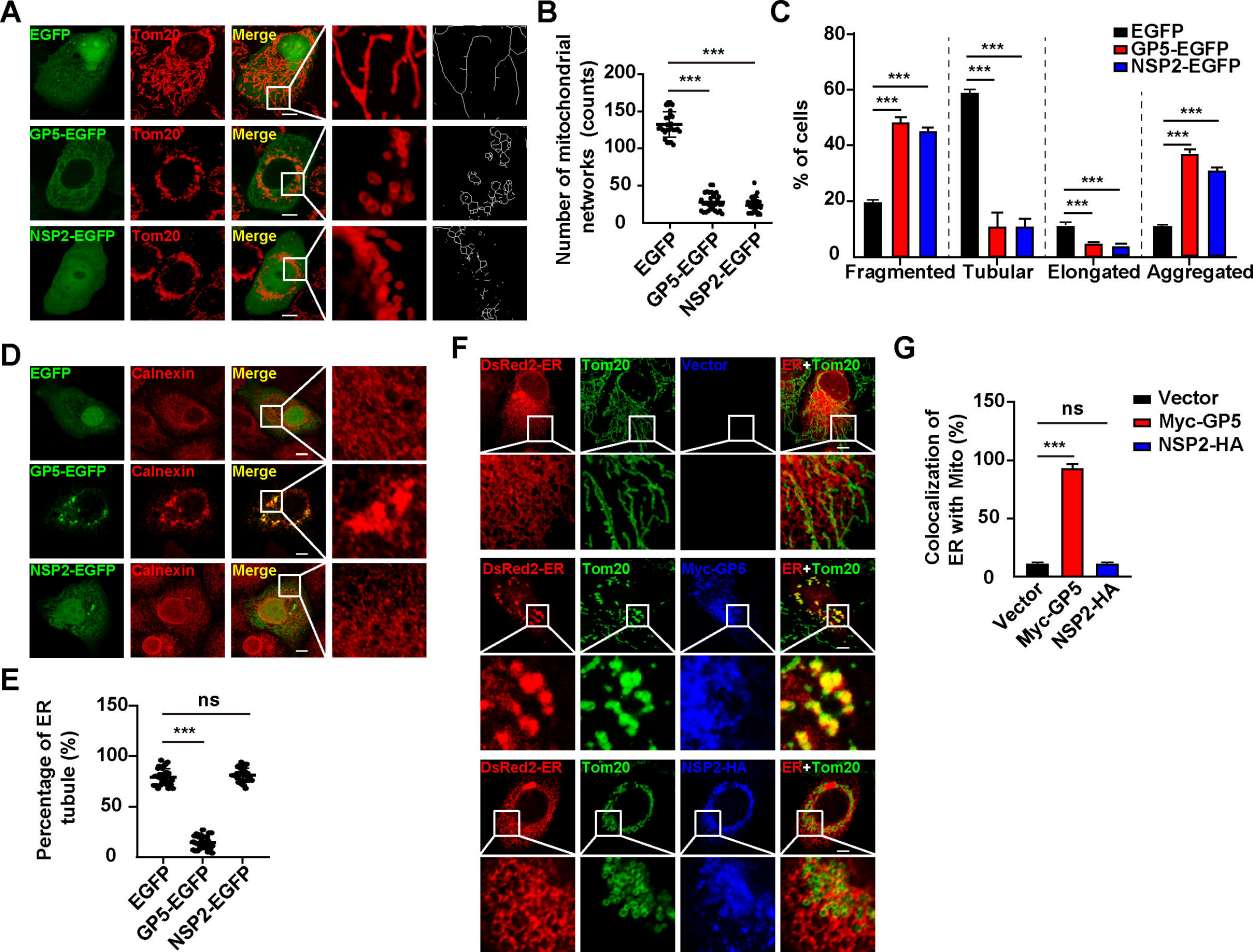
PRRSV GP5 is responsible for ER-mitochondria contact. (**A**) MARC-145 cells were transfected with EGFP, GP5-EGFP, or NSP2-EGFP plasmids for 24 h. The morphology of mitochondria (Tom20) was monitored by immunofluorescence analysis. Scale bar: 10 µm. (**B**) Quantification of mitochondrial networks number from (**A**) (*n* = 30). ^***^P < 0.001. (**C**) Quantification of the fragmented, tubular, elongated, and aggregated mitochondria from (**A**) (*n* = 30). ^***^P < 0.001. (**D**) MARC-145 cells were transfected with EGFP, GP5-EGFP, or NSP2-EGFP plasmids for 24 h. The morphology of ER (calnexin) was monitored by immunofluorescence analysis. Scale bar: 10 µm. (**E**) Quantification of the percentage of ER tubules from (**D**) (*n* = 30). ^***^P < 0.001. ns, no significance. (**F**) MARC-145 cells were co-transfected with pDsRed2-ER and vector, Myc-GP5, or NSP2-HA for 24 h. The co-localization of ER (DsRed) with mitochondria (Tom20) was monitored by immunofluorescence analysis. Scale bar: 10 µm. (**G**) Quantification of the co-localization of ER with mitochondria from (**F**) (*n* = 30). ^***^P < 0.001. ns, no significance.

To explore how PRRSV infection induces ER-mitochondria contact, we overexpressed PRRSV-encoded open reading frames (ORFs) in MARC-145 cells to identify the proteins that were involved in the alteration of the mitochondrial morphology. We observed that PRRSV ORF5 (GP5) and NSP2 could induce fragmented and aggregated structures in the mitochondria (Fig. 2A through C; Fig. S1); however, other PRRSV ORFs we tested did not have the same effect (Fig. S1). Mitochondrial networks, tubulation, and elongation were all affected either by GP5 or by NSP2 (Fig. 2B and C). Our findings were consistent with a previous report that PRRSV NSP2 alters mitochondrial morphology (28). We next examined the effect of GP5 and NSP2 on the morphology of ER. Immunofluorescence analysis of calnexin indicated that the ER became aggregated in GP5-expressing cells but not in cells that expressed NSP2 (Fig. 2D and E). These results indicated that GP5 could simultaneously alter mitochondrial and ER morphology. However, NSP2 could only result in aberrant mitochondrial morphology. To determine whether GP5 was involved in ER-mitochondria contact, we performed a co-localization assay of mitochondria with ER by immunofluorescence analysis. As anticipated, GP5 promoted the association of mitochondria with ER, whereas NSP2 failed (Fig. 2F and G). Altogether, our data demonstrated that PRRSV GP5 stimulated ER-mitochondria contact.

### PRRSV GP5 induces mitochondrial dysfunction

**Fig 3:**
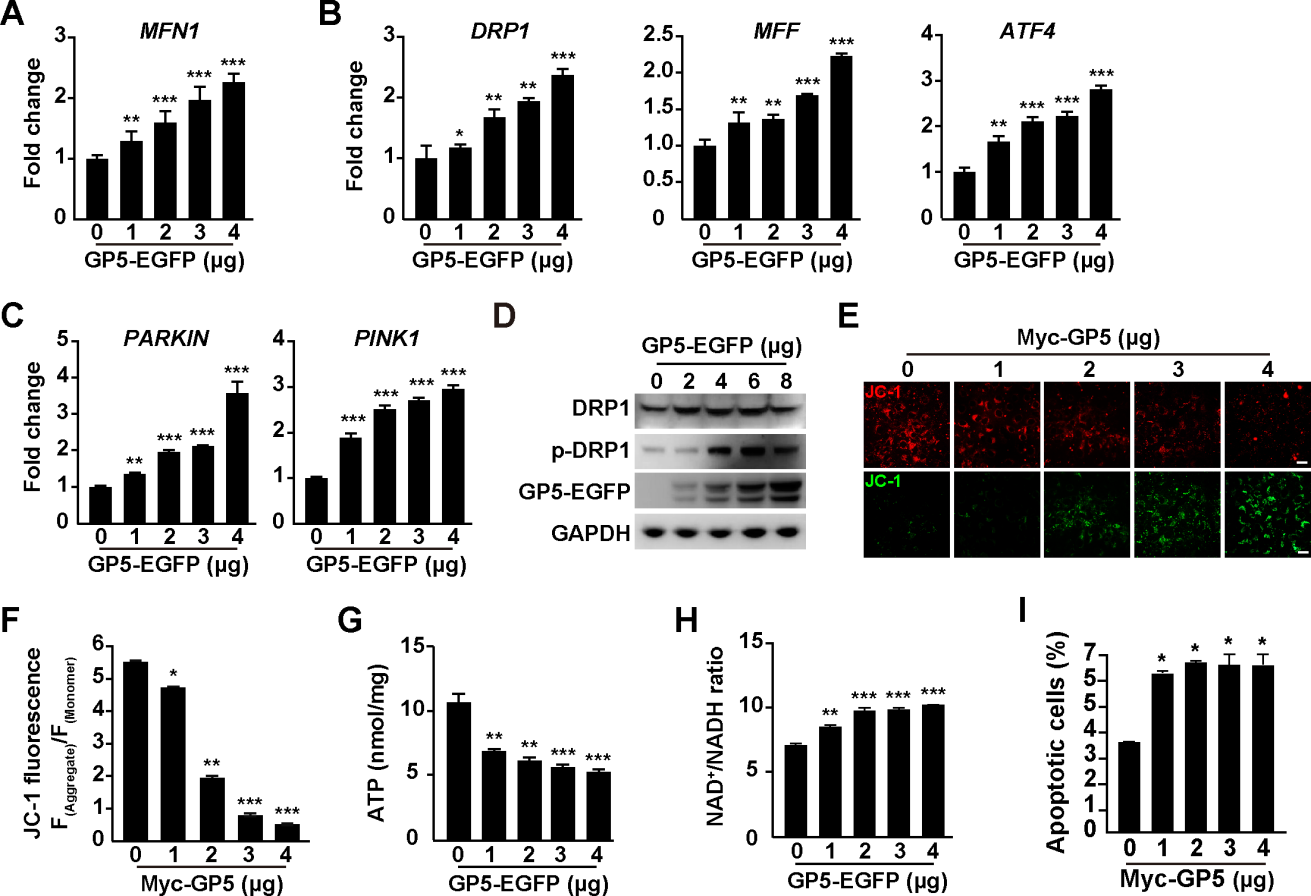
PRRSV GP5 alters mitochondrial dynamics. (**A**) MARC-145 cells were transfected with GP5-EGFP (0–4 µg) for 24 h. The mRNA levels of MFN1 were analyzed by qRT-PCR analysis. ^**^P < 0.01, ^***^P < 0.001. (**B**) MARC-145 cells were transfected with GP5-EGFP (0–4 µg) for 24 h. The mRNA levels of DRP1, MFF, and ATF4 were analyzed by qRT-PCR analysis. ^*^P < 0.05, ^**^P < 0.01, ^***^P < 0.001. (**C**) MARC-145 cells were transfected with GP5-EGFP (0–4 µg) for 24 h. The mRNA levels of PARKIN and PINK1 were analyzed by qRT-PCR analysis. ^**^P < 0.01, ^***^P < 0.001. (**D**) MARC-145 cells were transfected with GP5-EGFP (0–8 µg) for 24 h. DRP1, p-DRP1, Tom20, and GP5-EGFP were analyzed by immunoblotting analysis. (**E**) MARC-145 cells were transfected with Myc-GP5 (0–4 µg) for 24 h. The mitochondrial membrane potential was analyzed by JC-1 staining. Scale bar: 10 µm. (**F**) Quantification of the ratio of aggregated and monomer JC-1 from (**E**). ^*^P < 0.05, ^**^P < 0.01, ^***^P < 0.001. (**G**) Cellular ATP was measured in MARC-145 cells transfected with GP5-EGFP (0–4 µg) for 24 h. ^**^P < 0.01, ^***^P < 0.001. (**H**) NAD^+^/NADH ratio was measured in MARC-145 cells transfected with GP5-EGFP (0–4 µg) for 24 h. ^**^P < 0.01, ^***^P < 0.001. (**I**) MARC-145 cells were transfected with Myc-GP5 (0–4 µg) for 48 h. Apoptosis assay was performed by Annexin V FITC and PI staining. ^*^P < 0.05.

As our data suggested that PRRSV GP5 disrupted mitochondrial morphology, we sought to determine whether GP5 induced mitochondrial dysfunction. A quantitative real-time polymerase chain reaction (qRT-PCR) analysis indicated that the mRNA level of MFN1, which is involved in mitochondrial fusion (29), was upregulated by GP5 expression (Fig. 3A), and the expression of mitochondrial fission genes (30), such as *DRP1*, *MFF*, and *ATF4,* was also enhanced (Fig. 3B). Mitophagy is a cellular process that involves the selective degradation of damaged or unwanted mitochondria by autophagy (31). The mRNA levels of PARKIN and PINK1, which function together in mitophagy, were stimulated by GP5 (Fig. 3C). Furthermore, DRP1 phosphorylation is required for mitochondrial fission (32), and immunoblotting analysis indicated that GP5 expression promoted DRP1 phosphorylation (Fig. 3D). These results suggested that PRRSV GP5 promoted mitochondrial fusion, fission, and mitophagy.

JC-1 staining can be used to monitor changes in the Δψm in response to various stimuli. Under normal conditions, JC-1 forms aggregate and emit red fluorescence, indicating a high Δψm. However, in cells with a low Δψm, JC-1 remains in a monomeric form and emits green fluorescence (33). We next analyzed the Δψm by JC-1 staining. As shown in Fig. 3E, the red fluorescence of JC-1 declined gradually with increased GP5 expression. By measuring the ratio of red to green fluorescence, we found a decrease in the ratio of red/green fluorescence, indicating a loss of Δψm (Fig. 3F). However, the expression of NSP2 in cells did not influence Δψm, as indicated by unaltered red and green fluorescence and the red-to-green fluorescence ratio of JC-1 (Fig. S2A and B). Meanwhile, we measured ATP levels and NAD^+^ to NADH ratio and found that cellular ATP levels were lower and the ratio of NAD^+^/NADH was higher in GP5 transfected cells than in control cells (Fig. 3G and H). This suggested that GP5 disrupted oxidative phosphorylation in mitochondria; however, NSP2 exhibited no significant effect (Fig. S2C and D). Cell apoptosis assay indicated that GP5 induced apoptosis in MARC-145 cells (Fig. 3I) but NSP2 did not (Fig. S2E). Taken together, our data demonstrated that PRRSV GP5 induced mitochondrial dysfunction.

### PRRSV GP5 interacts with IP3R and VDAC1 to induce mitochondrial Ca^2+^ overload

**Fig 4:**
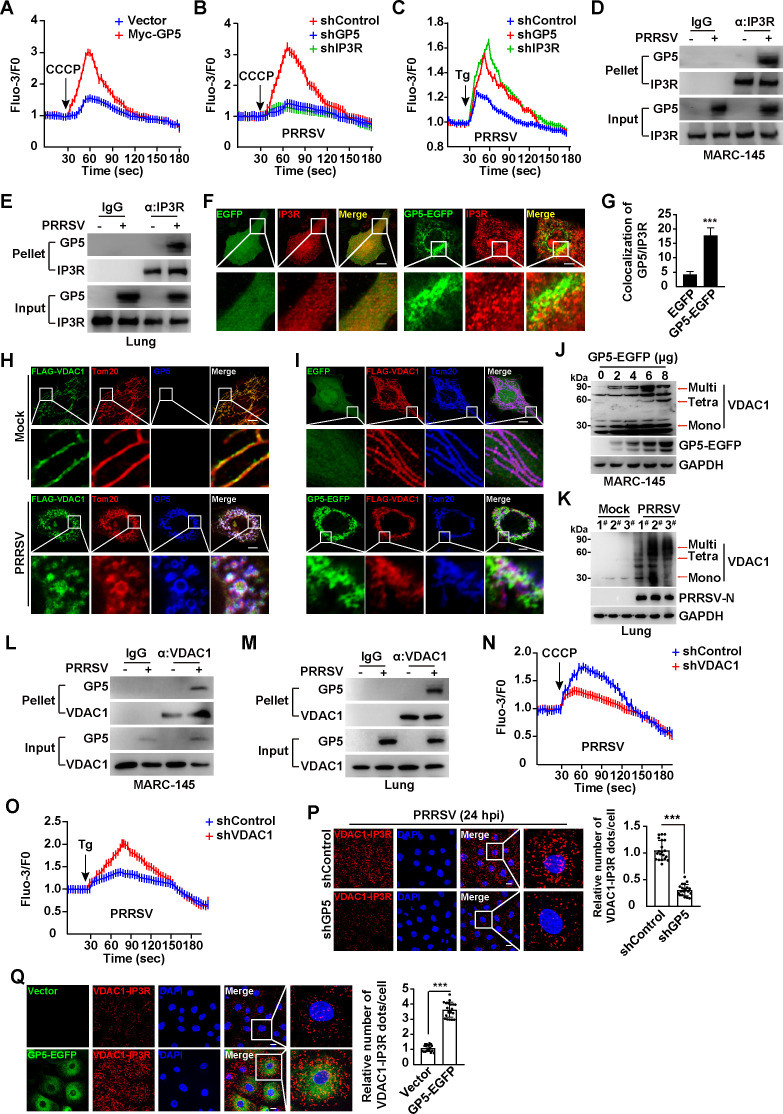
PRRSV GP5 interacts with IP3R and VDAC1 to induce Ca2^+^ efflux from ER into mitochondria. (**A**) MARC-145 cells were transfected with vector or Myc-GP5 for 24 h and then treated with CCCP (10 µM) to release mitochondrial Ca^2+^. (**B**) The shControl, shGP5, and shIP3R MARC-145 cells were infected with PRRSV (MOI = 1) for 24 h and then treated with CCCP (10 µM) to release mitochondrial Ca^2+^. (**C**) shControl, shGP5, and shIP3R MARC-145 cells were infected with PRRSV (MOI = 1) for 24 h and then treated with Tg (10 µM) to release ER Ca^2+^. (**D**) MARC-145 cells were mock-infected or infected with PRRSV (MOI = 1) for 24 h. The interaction of GP5 with IP3R was analyzed by Co-IP analysis. (**E**) The interaction of GP5 with IP3R was analyzed by Co-IP analysis in samples from mock-infected or PRRSV-infected porcine lungs. (**F**) MARC-145 cells transfected with EGFP-C1 and GP5-EGFP were assessed by immunostaining with antibody against IP3R (red). Scale bar, 10 µm. (**G**) Quantification of the co-localization of GP5-EGFP with IP3R from (**F**). ^***^P < 0.001. (**H**) MARC-145 cells were transfected with FLAG-VDAC1 for 24 h and then mock-infected or infected with PRRSV (MOI = 1) for 24 h. The co-localization of FLAG-VDAC1, GP5, and mitochondria (Tom20) was monitored by immunofluorescence analysis. Scale bar: 10 µm. (**I**) MARC-145 cells were co-transfected with FLAG-VDAC1 and EGFP or GP5-EGFP plasmids for 24 h. The co-localization of FLAG-VDAC1, GP5-EGFP, and Tom20 was monitored by immunofluorescence analysis. Scale bar: 10 µm. (**J**) MARC-145 cells were transfected with GP5-EGFP (0–8 µg) for 24 h. The VDAC1 oligomers and GP5-EGFP were analyzed by immunoblotting analysis. (**K**) The VDAC1 oligomers were analyzed by immunoblotting analysis in samples from mock-infected or PRRSV-infected porcine lungs. (**L**) MARC-145 cells were mock-infected or infected with PRRSV (MOI = 1) for 24 h. The interaction of GP5 with VDAC1 was analyzed by Co-IP analysis. (**M**) The interaction of GP5 with VDAC1 was analyzed by Co-IP analysis in samples from mock-infected or PRRSV-infected porcine lungs. (**N**) shControl and shVDAC1 MARC-145 cells were infected with PRRSV (MOI = 1) for 24 h and then treated with CCCP (10 µM) to release mitochondrial Ca^2+^. (**O**) shControl and shVDAC1 MARC-145 cells were infected with PRRSV (MOI = 1) for 24 h and then treated with Tg (10 µM) to release ER Ca^2+^. (**P**) shControl and shGP5 MARC-145 cells were infected with PRRSV (MOI = 1) for 24 h. The interaction of VDAC1 and IP3R was analyzed by PLA assay (left). Quantification of the relative number of VDAC1/IP3R dots per cell is shown on the right. Scale bar: 10 µm. ^***^P < 0.001. (**Q**) MARC-145 cells were transfected with vector or GP5-EGFP plasmid for 24 h. The interaction of VDAC1 and IP3R was analyzed by PLA assay (left). Quantification of the relative number of VDAC1/IP3R dots per cell is shown on the right. Scale bar: 10 µm. ^***^P < 0.001.

The ER and mitochondria play key roles in Ca^2+^ homeostasis (34). Mitochondrial Ca^2+^ overload can disrupt Δψm and impair oxidative phosphorylation. Because our data suggested that PRRSV GP5 affected Δψm and oxidative phosphorylation, we sought to determine whether GP5 promoted Ca^2+^ efflux from ER into mitochondria. MARC-145 cells were transfected with the control vector or Myc-GP5 for 24 h, and then mitochondrial Ca^2+^ was released by CCCP (35). As shown in Fig. 4A, excessive Ca^2+^ was detected from mitochondria in Myc-GP5 transfected cells, suggesting GP5 enhanced mitochondrial Ca^2+^. To identify which Ca^2+^ channel in the ER membrane was responsible for GP5-induced ER Ca^2+^ efflux, we performed the RNA interference to screen the ER Ca^2+^ channels. Seven known ER Ca^2+^ channels, including IP3R, RYR1, RYR2, RYR3, SERCA1, SERCA2, and SERCA3, were efficiently knocked-down in MARC-145 cells (Fig. S3A). Expression of GP5 in these cells suggested that CCCP failed to induce excessive mitochondria Ca^2+^ release in shIP3R MARC-145 cells, compared to the others (Fig. S3B). We then examined mitochondria and ER Ca^2+^ in MARC-145 cells with GP5 or IP3R knockdown during PRRSV infection (Fig. 4B and C; Fig. S3C and D). When Ca^2+^ was released from the mitochondria by CCCP, we observed decreased Ca^2+^ release in shGP5 and shIP3R cells compared to the control cells (Fig. 4B). Thapsigargin (Tg) is an inducer of ER Ca^2+^ release (36), and we treated shControl, shGP5, and shIP3R cells with Tg during PRRSV infection. We detected that PRRSV infection in shGP5 and shIP3R cells stimulated increases in Tg-induced ER Ca^2+^, suggesting interference of GP5 and IP3R resulted in the Ca^2+^ accumulation in the ER (Fig. 4C). These results suggested that GP5 facilitated IP3R-mediated ER Ca^2+^ efflux into mitochondria. We next determined whether GP5 interacted with IP3R. Co-immunoprecipitation (Co-IP) assay analysis indicated that GP5 was co-immunoprecipitated with IP3R either in MARC-145 cells or in PRRSV-infected lung tissues (Fig. 4D and E). Furthermore, increased co-localization between GP5-EGFP and IP3R was observed by immunofluorescence analysis, compared to that in control cells (Fig. 4F and G). These results suggested that GP5 interacted with IP3R to mediate ER Ca^2+^ efflux into mitochondria.

Voltage-dependent anion channel (VDAC) is a protein that is in the outer mitochondrial membrane and plays a key role in the transportation of Ca^2+^ into mitochondria (37). We observed that either PRRSV infection or GP5-EGFP expression could induce the aggregated VDAC1 structure, which co-localized with GP5 and Tom20 (Fig. 4H and I). Mitochondrial Ca^2+^ influx can enhance VDAC oligomer formation (38), so we performed a VDAC1 oligomerization assay. Transfection of GP5 in MARC-145 cells led to VDAC1 oligomerization (Fig. 4J). Induction of VDAC1 oligomer formation was also detected in PRRSV-infected lung tissues (Fig. 4K), suggesting that GP5 promoted VDAC1 oligomerization. To further identify the requirement of VDAC1 oligomerization for GP5-induced mitochondrial Ca^2+^ influx, we used the inhibitor NSC 15364 to prevent VDAC1 oligomerization (39). No obvious increases of mitochondrial Ca^2+^ release was detected in GP5-transfected cells in response to NSC 15364 treatment (Fig. S3E), indicating that VDAC1 oligomerization was essential for GP5-induced mitochondrial Ca^2+^ influx.

We then examined the interaction between GP5 and VDAC1 and found that GP5 and VDAC1 interacted with each other both in a cell culture model and in porcine lungs (Fig. 4L and M; Fig. S3F and G). CCCP-induced mitochondrial Ca^2+^ release was significantly lower in shVDAC1 than in control cells (Fig. 4N; Fig. S3H). In contrast, ER Ca^2+^ release induced by Tg was greater in shVDAC1 than in shControl cells (Fig. 4O). These results suggested that GP5 interacted with VDAC1 to promote VDAC1-dependent mitochondrial Ca^2+^ influx from the ER. To further confirm the complex formation of IP3R/GP5/VDAC1, we performed a proximity ligation assay (PLA). Compared to the control, cells with GP5 knockdown exhibited fewer numbers of VDAC1-IP3R dots during PRRSV infection (Fig. 4P). Increased red dots were detected in cells expressing GP5-EGFP (Fig. 4Q). Altogether, our data illustrated that PRRSV GP5 interacted with IP3R and VDAC1 to induce Ca^2+^ efflux from the ER into mitochondria.

### PRRSV GP5 elicits mROS and AMPK/mTOR/ULK1-dependent autophagy

**Fig 5:**
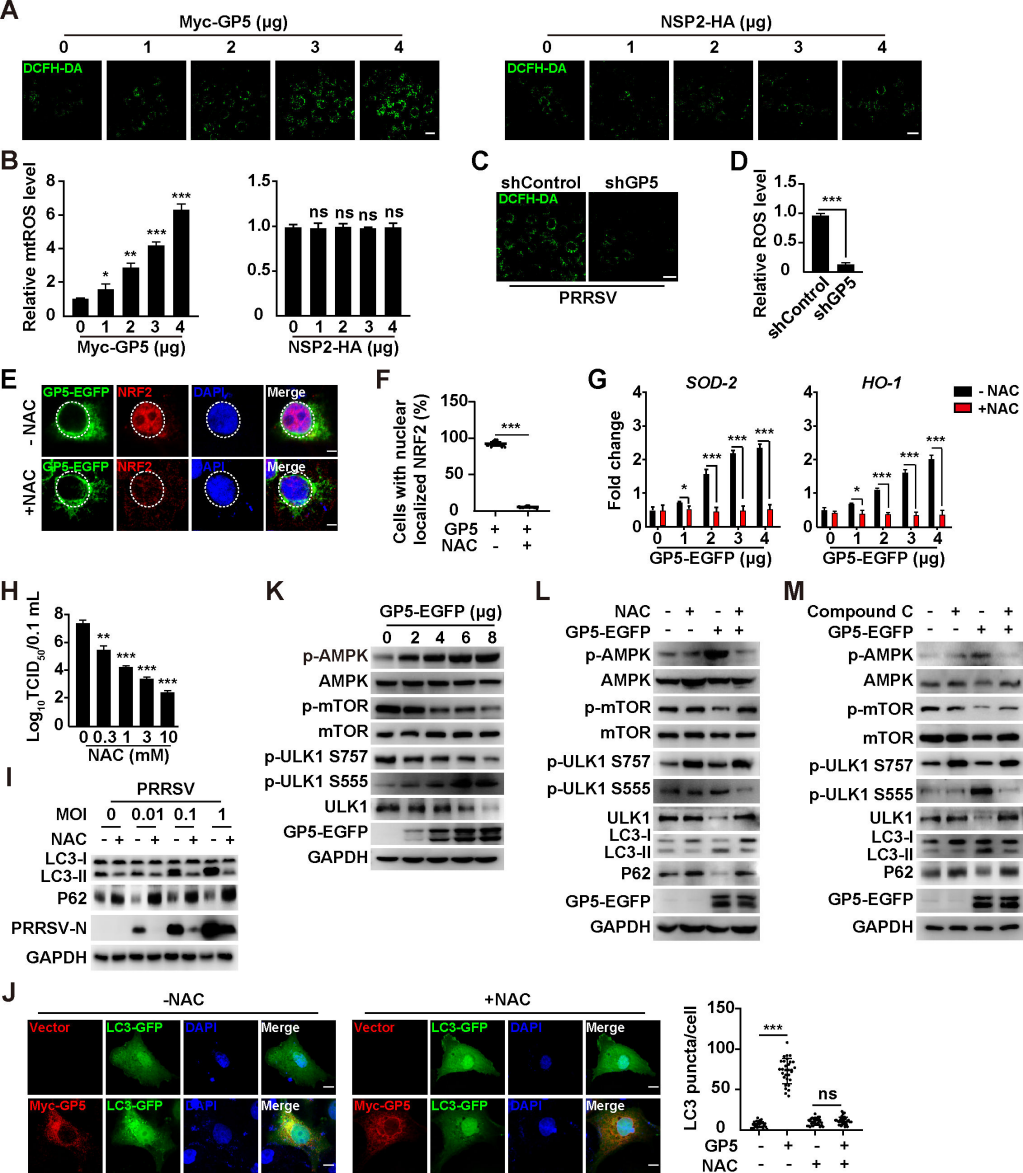
PRRSV GP5 induces ROS and activates autophagy through the AMPK/mTOR/ULK1 axis. (**A**) MARC-145 cells were transfected with GP5-EGFP (0–4 µg) or NSP2-HA (0–4 µg) for 24 h. Intracellular ROS was determined by DCFH-DA staining. Scale bar: 10 µm. (**B**) Quantification of the relative ROS levels from (**A**). ^*^P < 0.05, ^**^P < 0.01, ^***^P < 0.001. ns, no significance. (**C**) shControl and shGP5 MARC-145 cells were infected with PRRSV (MOI = 1) for 24 h. Intracellular ROS was determined by DCFH-DA staining. Scale bar: 10 µm. (**D**) Quantification of the relative ROS levels from (**C**). ^***^P < 0.001. (**E**) MARC-145 cells were transfected with GP5-EGFP (4 µg) and simultaneously treated with vehicle or NAC (10 mM) for 24 h. The subcellular localization of GP5-EGFP and NRF2 was detected by immunofluorescence analysis. Scale bar: 10 µm. (**F**) Quantification of cells with nuclear-localized NRF2 from (**E**). ^***^P < 0.001. (**G**) MARC-145 cells were transfected with GP5-EGFP (0–4 µg) and simultaneously treated with vehicle or NAC (10 mM) for 24 h. The mRNA levels of SOD-2 and HO-1 were analyzed by qRT-PCR analysis. ^*^P < 0.05, ^***^P < 0.001. (**H**) MARC-145 cells were infected with PRRSV (MOI = 1) and simultaneously treated with NAC (0–10 mM) for 48 h. Viral titers were assessed by TCID_50_ assay. ^**^P < 0.01, ^***^P < 0.001. (**I**) MARC-145 cells were infected with PRRSV (MOI = 0–1) and treated with NAC (10 mM) as indicated for 48 h. LC3-I, LC3-II, P62, PRRSV-N, and GAPDH were analyzed by immunoblotting analysis. (**J**) MARC-145 cells were co-transfected with Myc-GP5 and LC3-GFP and simultaneously treated with NAC (0–10 mM) as indicated for 24 h. LC3 puncta was detected by fluorescent microscopy (left). Quantification of LC3 puncta per cell is shown on the right. Scale bar: 10 µm. ^***^P < 0.001. ns, no significance. (**K**) MARC-145 cells were transfected with GP5-EGFP (0–8 µg) for 24 h. p-AMPK, AMPK, p-mTOR, mTOR, p-ULK1 S757, p-ULK1 S555, ULK1, GP5-EGFP, and GAPDH were analyzed by immunoblotting analysis. (**L**) MARC-145 cells were transfected with GP5-EGFP (8 µg) and treated with NAC (10 mM) for 24 h. p-AMPK, AMPK, p-mTOR, mTOR, p-ULK1 S757, p-ULK1 S555, ULK1, LC3-I, LC3-II, P62, GP5-EGFP, and GAPDH were analyzed by immunoblotting analysis. (**M**) MARC-145 cells were transfected with GP5-EGFP (4 µg) and treated with compound C (10 mM) for 24 h. p-AMPK, AMPK, p-mTOR, mTOR, p-ULK1 S757, p-ULK1 S555, ULK1, LC3-I, LC3-II, P62, GP5-EGFP, and GAPDH were analyzed by immunoblotting analysis.

We next attempted to determine whether PRRSV GP5 could induce mROS. The DCFH-DA assay is a common method for measuring ROS levels in cells (40). When MARC-145 cells were transfected with Myc-GP5, we observed an enhanced fluorescent signal of DCF, whereas NSP2 failed to induce any significant mROS production (Fig. 5A and B). Knockdown of GP5 significantly prevented mROS production during PRRSV infection (Fig. 5C and D). The nuclear factor erythroid 2-related factor 2 (NRF2) is a transcription factor that regulates the expression of genes involved in antioxidant defense. When ROS levels increase, NRF2 translocates into the nucleus and binds to antioxidant response elements in the promoter regions of target genes (41). Immunofluorescence analysis indicated that GP5 induced the translocation of NRF2 into the nucleus, which was abrogated by antioxidant NAC treatment (Fig. 5E and F). Furthermore, the NRF2-governed transcription of target genes, such as *SOD-2* and *HO-1*, was upregulated by GP5 transfection and inhibited by NAC (Fig. 5G). The production of infectious PRRSV progeny virus from NAC-treated cells was lower than in control cells (Fig. 5H). These results suggested that PRRSV GP5 elicited mROS to facilitate viral replication.

There is evidence to suggest that PRRSV induces autophagy to promote virus replication (42–44) and that ROS can modulate autophagy (45). Therefore, we sought to examine the role of GP5-induced mROS in autophagy. During PRRSV infection, NAC treatment decreased LC3-II expression and prevented P62 degradation (Fig. 5I). A significant increase of LC3 puncta was observed in Myc-GP5 expressing cells compared to control cells (Fig. 5J). NAC treatment abolished GP5-induced LC3 puncta formation (Fig. 5J). These results suggested that GP5 induced mROS to activate autophagy. It has been reported that ROS activated autophagy through the AMPK/mTOR/ULK1 pathway (46, 47), so we examined this pathway by immunoblotting analysis. As shown in Fig. 5K, GP5 led to the enhanced phosphorylation of AMPK and ULK1 (S555) and reduced phosphorylation of mTOR and ULK1 (S757), suggesting that GP5 activated AMPK to inhibit mTOR and induce ULK1-mediated autophagy. Treatment of cells with NAC or AMPK inhibitor compound C suppressed GP5-induced AMPK activation, mTOR inhibition, and autophagy induction, as indicated by the immunoblotting of phosphorylated AMPK, mTOR and ULK1, and LC3-II and P62 (Fig. 5L and M). Taken together, our data indicated that PRRSV GP5 elicited mROS/AMPK/mTOR/ULK1-dependent autophagy.

### PRRSV GP5-induced mROS activates NLRP3 inflammasome

**Fig 6:**
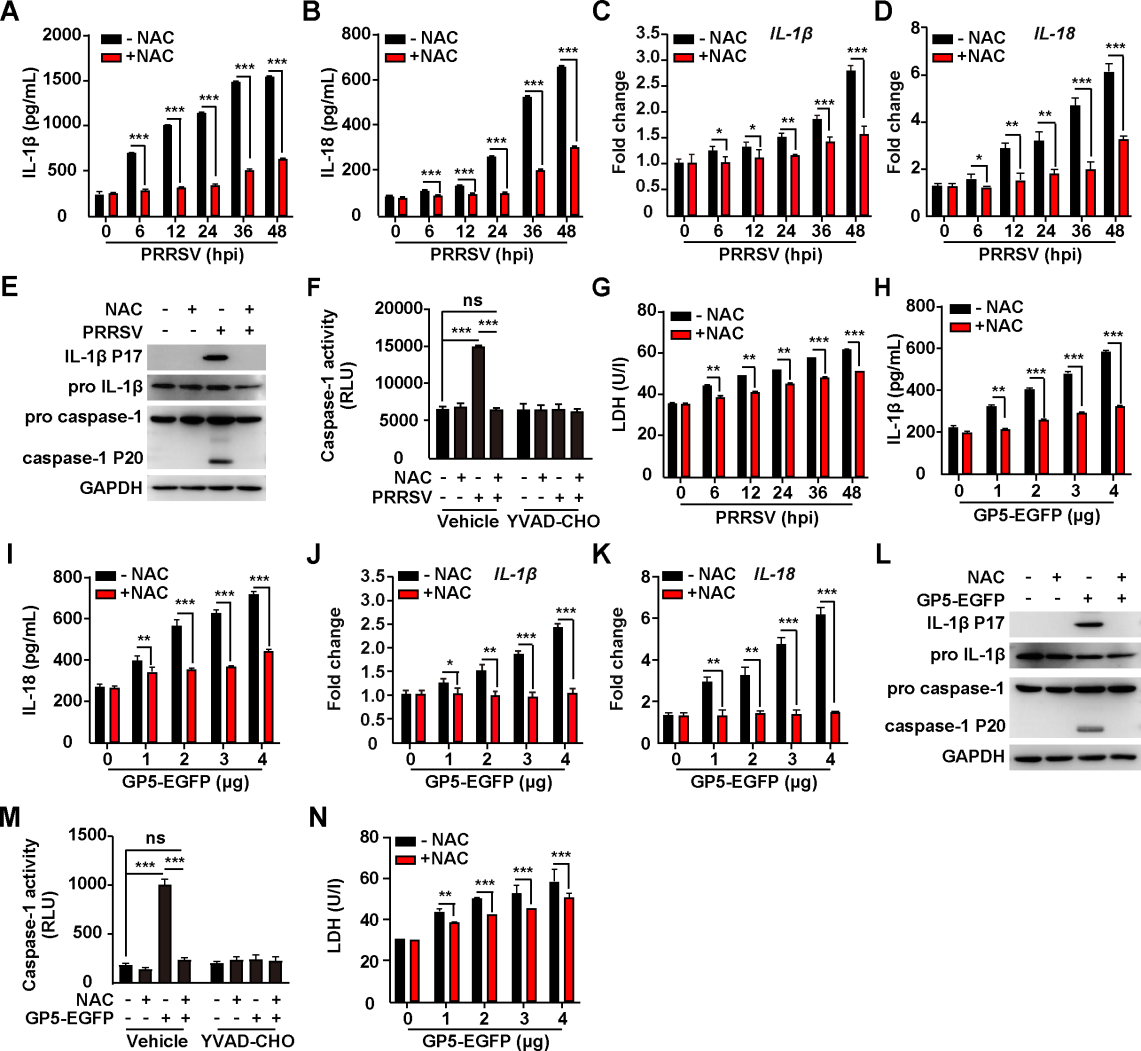
PRRSV GP5-induced ROS activates NLRP3 inflammasome. (**A, B**) iPAMs were infected with PRRSV (MOI = 1) simultaneously treated with NAC (10 mM) as indicated for 0–48 h. IL-1β (**A**) and IL-18 (**B**) in the medium were measured by ELISA. ^***^P < 0.001. (**C, D**) iPAMs were infected with PRRSV (MOI = 1) and simultaneously treated with NAC (10 mM) as indicated for 0–48 h. The mRNA levels of IL-1β (**C**) and IL-18 (**D**) were analyzed by qRT-PCR analysis. ^*^P < 0.05, ^**^P < 0.01, ^***^P < 0.001. (**E**) iPAMs were infected with PRRSV (MOI = 1) and simultaneously treated with NAC (10 mM) as indicated for 48 h. IL-1β, pro-IL-1β, pro-caspase-1, caspase-1 P20, and GAPDH were analyzed by immunoblotting analysis. (**F**) Caspase-1 activity was assessed in iPAMs infected with PRRSV (MOI = 1) and simultaneously treated with NAC (10 mM) or YVAD-CHO (5 µM) as indicated for 24 h. ^***^P < 0.001. ns, no significance. (**G**) The LDH activities were measured in the medium from iPAMs infected with PRRSV (MOI = 1) and simultaneously treated with NAC (10 mM) as indicated for 0–48 h. ^**^P < 0.01, ^***^P < 0.001. (**H, I**) iPAMs were transfected with GP5-EGFP (0–4 µg) and simultaneously treated with NAC (10 mM) as indicated for 24 h. IL-1β (**H**) and IL-18 (**I**) in the medium were measured by ELISA. ^**^P < 0.01, ^***^P < 0.001. (**J, K**) iPAMs were transfected with GP5-EGFP (0–4 µg) and simultaneously treated with NAC (10 mM) as indicated for 24 h. The mRNA levels of IL-1β (**J**) and IL-18 (**K**) were analyzed by qRT-PCR analysis. ^*^P < 0.05, ^**^P < 0.01, ^***^P < 0.001. (**L**) iPAMs were transfected with GP5-EGFP (0–4 µg) and simultaneously treated with NAC (10 mM) as indicated for 24 h. IL-1β, pro-IL-1β, pro-caspase-1, caspase-1 P20, and GAPDH were analyzed by immunoblotting analysis. (**M**) Caspase-1 activity was assessed in iPAMs cells transfected with GP5-EGFP (4 µg) and simultaneously treated with NAC (10 mM) or YVAD-CHO (5 µM) as indicated for 24 h. ^***^P < 0.001. ns, no significance. (**N**) The LDH activities were measured in the medium from iPAMs transfected with GP5-EGFP (0–4 µg) and simultaneously treated with NAC (10 mM) as indicated for 24 h. ^**^P < 0.01, ^***^P < 0.001.

ROS can activate the NLRP3 inflammasome, leading to the production of pro-inflammatory cytokines, such as IL-1β and IL18 (48). An enzyme-linked immunosorbent (ELISA) indicated that the enhanced secretion of IL-1β and IL18 was detected in the medium of iPAMs infected with PRRSV (Fig. 6A and B). PRRSV infection also upregulated the transcription of *IL-1β* and *IL18* (Fig. 6C and D). NAC treatment suppressed PRRSV-induced secretion and transcription of IL-1β and IL18 (Fig. 6A through D). Pro-IL-1β and caspase-1 need to be cleaved to IL-1β P17 and caspase-1 P20 for their maturation. We observed that the cleavage of IL-1β and caspase-1 occurred in PRRSV-infected iPAMs but did not occur after treatment with NAC (Fig. 6E). During PRRSV infection, the enhanced caspase-1 activity was inhibited either by NAC or by caspase-1 inhibitor YVAD-CHO (Fig. 6F). LDH secretion is a hallmark of NLRP3 inflammasome-induced pyroptosis (49). As shown in Fig. 6G, PRRSV infection promoted LDH release, which was inhibited by NAC. These results suggested that PRRSV infection activated NLRP3 inflammasome through mROS.

We next examined the role of PRRSV GP5 in the activation of NLRP3 inflammasome. We transfected iPAMs with GP5-EGFP and measured IL-1β and IL18 secretion. Similar to in PRRSV-infected iPAMs, GP5 expression stimulated the release of IL-1β and IL18, whereas NAC inhibited this effect (Fig. 6H and I). In GP5-GFP expressing iPAMs, the mRNA levels of IL-1β and IL18 were significantly higher in control than in NAC-treated iPAMs (Fig. 6J and K). Mature IL-1β P17 and caspase-1 P20 were only detected in GP5-transfected but not in NAC-treated iPAMs (Fig. 6L). NAC and YVAD-CHO suppressed GP5-activated caspase-1 activity (Fig. 6M). The LDH activity in the medium was significantly enhanced in the control compared with NAC-treated iPAMs (Fig. 6N). In addition, knockdown of GP5 inhibited PRRSV-induced activation of NLRP3 inflammasome, as indicated by decreased transcription of IL-1β and IL18 mRNA from shGP5 iPAM cells, compared to that from shControl cells (Fig. S4A and B). Altogether, our data demonstrated that PRRSV ORF5 activated NLRP3 inflammasome through mROS.

### PRRSV ORF5-activated autophagy antagonizes NLRP3 inflammasome

**Fig 7:**
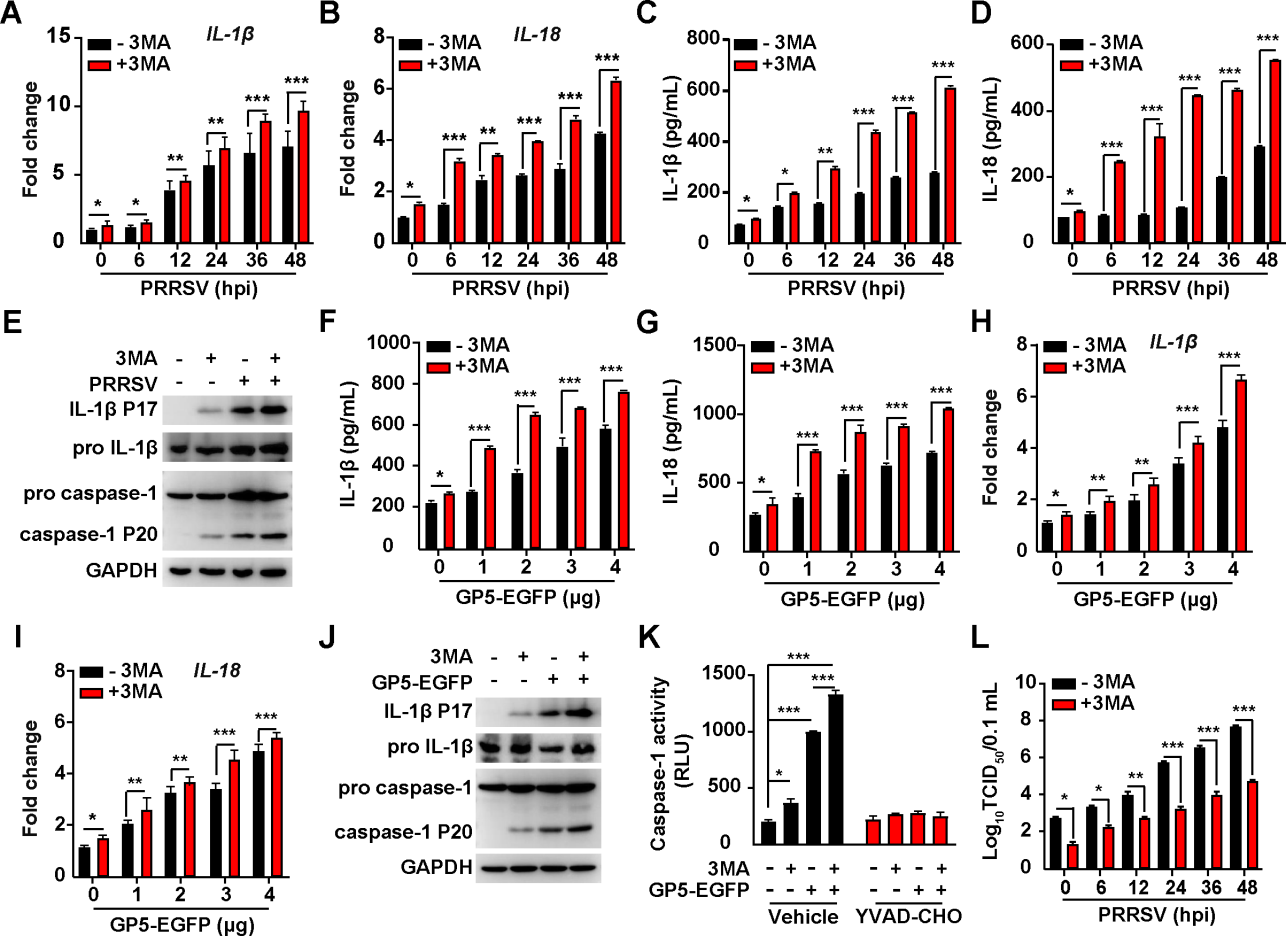
PRRSV GP5-induced autophagy antagonizes NLRP3 inflammasome. (**A, B**) The iPAMs were infected with PRRSV (MOI = 1) and simultaneously treated with 3MA (10 µM) as indicated for 0–48 h. The mRNA levels of IL-1β (**A**) and IL-18 (**B**) were analyzed by qRT-PCR analysis. ^*^P < 0.05, ^**^P < 0.01, ^***^P < 0.001. (**C, D**) iPAMs were infected with PRRSV (MOI = 1) simultaneously treated with 3MA (10 µM) as indicated for 0–48 h and IL-1β (**C**) and IL-18 (**D**) in the medium were measured by ELISA. ^*^P < 0.05, ^**^P < 0.01, ^***^P < 0.001. (**E**) iPAMs were infected with PRRSV (MOI = 1) and simultaneously treated with 3MA (10 µM) as indicated for 48 h. IL-1β, pro-IL-1β, pro-caspase-1, caspase-1 P20, and GAPDH were analyzed by immunoblotting analysis. (**F, G**) iPAMs were transfected with GP5-EGFP (0–4 µg) and simultaneously treated with 3MA (10 µM) as indicated for 24 h. IL-1β (**F**) and IL-18 (**G**) in the medium were measured by ELISA. ^*^P < 0.05, ^***^P < 0.001. (**H, I**) iPAMs were transfected with GP5-EGFP (0–4 µg) and simultaneously treated with 3MA (10 µM) as indicated for 24 h. The mRNA levels of IL-1β (**H**) and IL-18 (**I**) were analyzed by qRT-PCR analysis. ^*^P < 0.05, ^**^P < 0.01, ^***^P < 0.001. (**J**) iPAMs were transfected with GP5-EGFP (4 µg) and simultaneously treated with 3MA (10 µM) as indicated for 24 h. IL-1β, pro-IL-1β, pro-caspase-1, caspase-1 P20, and GAPDH were analyzed by immunoblotting analysis. (**K**) Caspase-1 activity was assessed in iPAMs transfected with GP5-EGFP (4 µg) and simultaneously treated with 3MA (10 µM) as indicated for 24 h. ^*^P < 0.05, ^***^P < 0.001. (**L**) iPAMs were infected with PRRSV (MOI = 1) and simultaneously treated with 3MA (10 µM) as indicated for 0–48 h. Viral titers were assessed by TCID_50_ assay. ^*^P < 0.05, ^**^P < 0.01, ^***^P < 0.001.

Owing to the sophisticated roles of autophagy in regulating the inflammasome (50, 51), we set out to analyze the interaction between PRRSV ORF5-activated autophagy and NLRP3 inflammasome. We inhibited autophagy by 3MA and analyzed NLRP3 inflammasome activation in response to PRRSV infection or GP5 expression. As shown in Fig. 7A and B, iPAMs treated with 3MA exhibited increased mRNA levels of IL-1β and IL18 during PRRSV infection, compared to control cells. Consistently, secretion of IL-1β and IL18 was also enhanced by 3MA treatment (Fig. 7C and D). The maturation of IL-1β P17 and caspase-1 P20 was further stimulated when iPAMs were infected with PRRSV and treated with 3MA (Fig. 7E). These results suggested that autophagy induced by PRRSV infection alleviated NLRP3 inflammasome activation.

We finally examined the role of PRRSV GP5-induced autophagy in modulating NLRP3 inflammasome. Although GP5 stimulated the secretion and transcription of IL-1β and IL18, 3MA treatment augmented this effect (Fig. 7F through I). The increased maturation of IL-1β and caspase-1 was detected by the immunoblotting analysis of IL-1β P17 and caspase-1 P20 when iPAMs were transfected with GP5-EGFP and treated with 3MA (Fig. 7J). Inhibition of autophagy by 3MA enhanced the caspase-1 activity in GP5-transfected iPAMs (Fig. 7K). A viral titer assay indicated that 3MA suppressed PRRSV proliferation in a time-dependent manner (Fig. 7L). Taken together, our data indicated that PRRSV ORF5-activated autophagy antagonized NLRP3 inflammasome to facilitate viral replication.

## DISCUSSION

**Fig 8:**
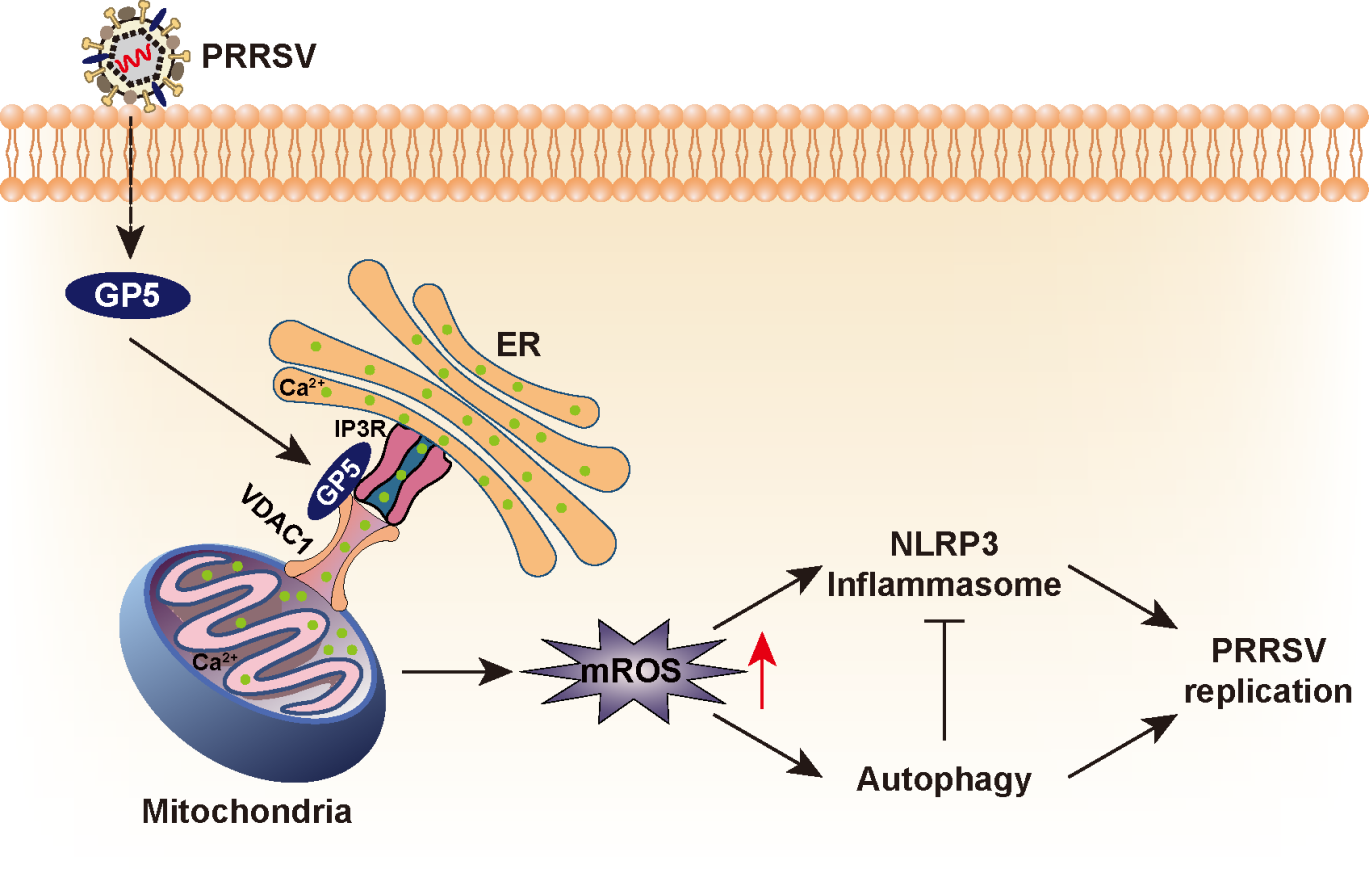
A schematic model showing PRRSV GP5 stimulates mROS to facilitate viral replication.

Here, we report that PRRSV hijacks ER-mitochondria contact to induce mROS for optimal viral replication. PRRSV ORF5 interacts with IP3R and VDAC1 to promote excessive mitochondrial Ca^2+^ uptake from the ER to induce mitochondrial dysfunction and mROS production. Elevated mROS activates AMPK/mTOR/ULK1-dependent autophagy and triggers NLRP3 inflammasome. Autophagy alleviates NLRP3 inflammasome-mediated antiviral innate immunity that assists viral replication (Fig. 8).

Although a previous study has suggested that PRRSV NSP2 alters mitochondrial morphology (28), our data indicated that NSP2 had no effect on mitochondrial dysfunction and mROS production. We have demonstrated that PRRSV GP5 could simultaneously induce morphological alteration in the ER and mitochondria and could promote MAM formation through the interaction between IP3R and VDAC1. Several proteins are involved in mediating the communication and interaction between ER and mitochondria (52). VAMP-associated proteins are ER-resident proteins that interact with a family of proteins called mitofusins on the outer mitochondrial membrane. This interaction helps tether the ER and mitochondria together (53). The Sigma-1 receptor is an ER-resident protein acting as a chaperone and interacts with various proteins, including IP3Rs and VDACs (54). GRP75 is primarily known as a mitochondrial chaperone protein. It interacts with IP3R and may play a role in regulating Ca^2+^ transfer between the ER and mitochondria (55). Whether GP5-mediated ER-mitochondria contact requires these proteins, such as mitofusins and GRP75, has not been investigated in our present study. The precise mechanisms and interactions between these proteins are complex and vary depending on the cellular context and specific cellular functions. A better understanding of how GP5 interacts with proteins involved in MAM formation will be an advantage in the development of anti-PRRSV therapeutics.

ROS play a complex role in viral infections (56). When infected with a virus, host cells produce ROS as a defense mechanism to eliminate the invading virus. ROS can activate signaling pathways, such as NLRP3 inflammasome, that lead to the production of antiviral cytokines and chemokines, promoting an inflammatory response and recruiting immune cells to the site of infection. However, some viruses can induce an increase in ROS production, which can serve as a signaling mechanism to activate viral replication or modulate host cell signaling pathways to promote viral survival and spread. For example, the influenza A virus NS1 protein can inhibit the production of ROS and suppress host antiviral responses (57). HCV induces the production of ROS through multiple mechanisms, including the activation of NADPH oxidases and the disruption of mitochondrial function (58). The manipulation of ROS levels by herpesviruses has been implicated in viral replication, modulation of host cell signaling pathways, and promotion of viral pathogenesis (59). Our data indicated that PRRSV GP5 induced mROS to promote viral replication. The treatment of cells with antioxidant NAC significantly inhibited PRRSV replication. Ours and previous examples highlight the diverse strategies employed by different viruses in their interactions with ROS.

Autophagy is a cellular process involved in the degradation and recycling of damaged or unnecessary cellular components, such as proteins, organelles, and invading pathogens (60). During viral infection, autophagy plays a dual role; it can either have antiviral functions, where it targets and degrades viral components, or it can be manipulated by the virus to benefit viral replication and spread. Autophagy can provide a source of lipids and membranes required for HCV replication, and it can also suppress the host’s immune response against HCV (61). Dengue virus uses the autophagy machinery to promote its replication and to evade the host immune system (62). Our previous study has suggested that PRRSV activated autophagy to stimulate lipolysis (42). Several reports have indicated that autophagy is beneficial in PRRSV replication. PRRSV NSP2 co-localized with LC3 in MARC-145 cells may induce autophagy to promote viral replication (44). PRRSV triggers mitochondrial fission and mitophagy to attenuate apoptosis that facilitates viral replication (28). Diao and colleagues indicated that PRRSV infection activated autophagy via ER stress-induced Ca^2+^ signaling to facilitate virus replication (63). We revealed that PRRSV GP5 altered ER morphology and induced ER Ca^2+^ efflux. Depletion of Ca^2+^ from the ER leads to ER stress due to impaired folding capacity and subsequent accumulation of misfolded or unfolded proteins within the ER lumen (64, 65). Therefore, we hypothesized that PRRSV GP5 might induce ER stress.

ROS have been implicated in the activation of NLRP3 inflammasome. Our data indicated that NLRP3 inflammasome were activated by GP5-induced mROS. It has been suggested that DDX19A senses the PRRSV genome and mediates NLRP3-dependent inflammasome activation (66). PRRSV infection induces gasdermin D-driven pyroptosis of porcine alveolar macrophages through NLRP3 inflammasome activation (67). Recently, Li and colleagues have reported that PRRSV infection activated the NLRP3 inflammasome by inducing cytosolic mitochondrial DNA stress (68). Mitochondrial DNA is believed to be a danger-associated molecular pattern that can activate NLRP3 inflammasome (69). Given the observations that GP5 led to mitochondrial dysfunction, we hypothesized that GP5-induced mitochondrial DNA leakage is an inducer of NLRP3 inflammasome activation, as well as mROS (70). We have further indicated that autophagy counteracts NLRP3 inflammasome activation to promote PRRSV replication. Our research has demonstrated that GP5-induced autophagy played a dual role in PRRSV replication. On the one hand, GP5 triggered autophagy to promote lipophagy and, on the other hand, autophagy inhibited NLRP3 inflammasome to evade antiviral innate immunity.

## MATERIALS AND METHODS

### Pigs

Thirty-day-old specific-pathogen-free weaned piglets (free of PRRSV, pseudorabies virus, porcine circovirus, and classical swine fever virus) were challenged intranasally with PRRSV (2 × 10^5^ TCID_50_/piglet) and were sacrificed at 30 days post-infection. The lung tissues were collected and immediately snap-frozen in liquid nitrogen and stored at −80°C.

### Cells and virus

MARC-145, HEK293T, and iPAMs cells (immortalized from porcine alveolar macrophages) were grown in monolayers at 37°C under 5% CO_2_ and maintained in Dulbecco’s modified Eagle’s medium (DMEM; 10566-016; GIBCO) supplemented with 10% fetal bovine serum (FBS; 10099141C; GIBCO), 100 units/mL penicillin, and 100 µg/mL streptomycin sulfate (Sangon; B540732). PRRSV (strain HN07-1, accession number KX766378.1) was used as described previously (42).

### Chemicals and antibodies

N-acetylcysteine (NAC, IA0050) was purchased from Solarbio; 3-Methyladenine (3MA, HY-19312), CCCP (HY-100941), NSC 15364 (HY-108937), and compound C (HY-13418A) were purchased from MedChemExpress; JC-1 (C2006) and 2,7-dichlorofluorescin diacetate (DCFH-DA, S0033S) were purchased from Beyotime; Thapsigargin (Tg, GC11482) was purchased from GLPBIO; YVAD-CHO (G9951) was purchased from Promega; Fluo-3-AM (F1242) was purchased from Thermo Fisher Scientific.

Anti-p-DRP1 (#3455S), anti-AMPK (#2532S), anti-p-AMPK (#50,081 s), anti-mTOR (#2983T), anti-p-mTOR (#5536T), anti-ULK1 (#6439S), anti-p-ULK1 (ser757) (#14,202T), anti-p-ULK1(ser555) (#5869T), anti-p-DRP1 (#3455S), anti-EGFP (#2956), anti-LAMP1 (#9091T), anti-P62 (#5114), anti-NRF2 (#12,721s) antibodies were purchased from Cell Signaling Technology; anti-IL-1β was from R&D Systems (AF-401-NA); anti-caspase-1 was from Santa Cruz Biotechnology (sc-398715); anti-Myc (AB0001) was purchased from Abways; anti-GM130 (11308-1-AP), anti-Tom20 (11802-1-AP), anti-VDAC1 (55259-1-AP), anti-LC3 (14600-1-AP), and GAPDH (10494-1-AP) antibodies were purchased from Proteintech; anti-HA (A01244-100) antibody was purchased from GenScript; anti-calnexin (C4731), anti-FLAG (F1804), and anti-IP3R (F9291) antibodies were purchased from Sigma-Aldrich; horseradish-peroxidase (HRP)-conjugated goat anti-rabbit IgG (A16104), HRP-conjugated goat anti-mouse (PA1-74421) IgG, Alexa-Fluor-488-conjugated goat anti-rabbit IgG (A11034), Alexa-Fluor-568-conjugated goat anti-mouse IgG (A11004), and Alexa-Fluor-633-conjugated goat anti-mouse IgG (A-21052) were purchased from Thermo Fisher Scientific. Antiserum against PRRSV GP5 and N was generated by immunization of mice with purified recombinant GP5 and N, respectively.

### Plasmids and transfection

The plasmids pDsRed2-ER (#55836), pmCherry-mito (#55102), pEGFP-LC3 (24920), pEGFP-C1 (#28235), and pN-5 × Myc (#131244) were from Addgene; p3 × FLAG-CMV-10 (E4401) was from Sigma-Aldrich.

The coding sequences of PRRSV ORF2α, ORF2β, ORF3, ORF4, ORF5, ORF6, ORF7, NSP1, NSP1α, NSP1β, NSP2, NSP3, NSP4, NSP5, NSP6, NSP7, NSP7α, NSP8, NSP9, NSP10, NSP11, and NSP12 were amplified from PRRSV genomic cDNA and cloned into pEGFP-C1. The coding sequence of VDAC1 was amplified from the cDNA of HEK293 cells and cloned into p3 × FLAG-CMV-10 (FLAG-VDAC1), pCDNA3.0-HA (HA-VDAC1), or pN-5 × Myc. All plasmids were transfected with Lipofectamine 3000 (L3000015, Invitrogen) according to the manufacturer’s instructions.

### qRT-PCR analysis

Total RNA was isolated with TRIzol Reagent (9108, TaKaRa) and subjected to cDNA synthesis using a PrimeScript RT reagent kit (RR047A, TaKaRa). The qRT-PCR was performed in triplicate using SYBR Premix Ex Taq (RR820A, TaKaRa) according to the manufacturer’s instructions, and data were normalized to the level of *ACTB* expression in each sample. Melting curve analysis indicated the formation of a single product in all cases. The 2^−ΔΔ*Ct*^ method was used to calculate changes in relative expression. Primers used for qRT-PCR were shown in Table S1.

### Immunoblotting analysis

Cells were harvested and lysed with lysis buffer (50 mM Tris-HCl pH 8.0, 150 mM NaCl, 1% Triton X-100, 1% sodium deoxycholate, 0.1% SDS, and 2 mM MgCl_2_) supplemented with a protease and phosphatase inhibitor cocktail (HY-K0010 and HY-K0022, MedChemExpress). Protein samples were separated by SDS-PAGE and then transferred to a membrane (ISEQ00010, Millipore), which was incubated in 5% nonfat milk for 1 h at room temperature. The membrane was then incubated with primary antibody overnight at 4°C, followed by HRP-conjugated secondary antibody for 1 h at room temperature. Immunoblotting results were visualized with Luminata Crescendo Western HRP substrate (WBLUR0500, Millipore) on a GE AI600 imaging system.

### Immunofluorescence analysis

MARC-145 cells cultured on coverslips (12-545-80, Thermo Fisher Scientific) in 12-well plates were fixed with 4% (wt/vol) paraformaldehyde at room temperature for 20 min. After washing three times with PBS, cells were permeabilized with 0.2% Triton X-100 for 20 min and then blocked with 10% FBS. The specific primary antibodies diluted in 10% FBS were added to the cells and incubated for 1 h at room temperature. After washing three times with PBS, cells were incubated with appropriate secondary antibodies diluted in 10% FBS for 1 h at room temperature. The cells were washed in PBS and mounted in ProLong Diamond with DAPI (#P36971, Invitrogen). Images were captured on a Zeiss LSM 800 confocal microscope and processed in ImageJ software for quantitative image analysis.

### Apoptosis assay

MARC-145 cells were transfected with Myc-GP5 (0–4 µg) or NSP2-HA (0–4 µg) for 48 h. Annexin V/PI staining was performed with a Dead Cell Apoptosis Kit with Annexin V FITC and PI (V13242, ThermoFisher) according to the manufacturer’s instructions. The percentage of dead cells (positive for both Annexin V and PI) was measured by flow cytometry on a CytoFLEX instrument (BECKMAN COULTER).

### VDAC1 oligomerization assay

Cells or lung tissues were lysed in homogenization buffer (20 mM HEPES-KOH, pH 7.5, 10 mM KCl, 1.5 mM MgCl_2_, 1 mM EDTA, 1 mM EGTA, and 320 mM sucrose) supplemented with a protease and phosphatase inhibitor cocktail (MedChemExpress) via passage through a 21-gage needle 30 times. The lysates were subjected to centrifugation at 1,500 × *g* for 10 min. The supernatants were diluted with 1 vol of CHAPS buffer (20 mM HEPES-KOH, pH 7.5, 5 mM MgCl_2_, 0.5 mM EGTA, and 0.1% CHAPS) supplemented with a protease and phosphatase inhibitor cocktail (MedChemExpress) and centrifuged at 5,000 × *g* for 10 min. The pellets were washed three times with ice-cold PBS and then resuspended in 30 mL of CHAPS buffer supplemented with 2 mM DSS crosslinker (21655, Thermo Fisher Scientific). After incubation at 37°C for 20 min, the reaction was quenched by the addition of LaemmLi buffer, and the samples were subjected to SDS-PAGE.

### Dichloro-dihydro-fluorescein diacetate assay

Dichloro-dihydro-fluorescein diacetate (DCFH-DA) (2',7'-dichlorofluorescin diacetate) is a non-fluorescent dye that can be taken up by cells and converted into the fluorescent dye dichlorofluorescein (DCF) in the presence of ROS. The fluorescence intensity of DCF can then be measured by fluorescence microscopy to determine the level of ROS in the cells. After treatment, cells were incubated with DCFH-DA (10 µM) in the dark at 37°C for 30 min and then rinsed twice with PBS. Images were taken using a fluorescent microscope and the appropriate bandpass FITC filter. The relative fluorescence was measured using ImageJ software.

### RNA interference

Short hairpin RNAs (Table S2) were synthesized as double-stranded oligonucleotides and cloned into the pLKO.1 vector (#10878, Addgene) and co-transfected with packaging plasmids pMD2.G (#12259, Addgene) and psPAX2 (#12260, Addgene) into HEK293T cells. Lentiviruses were harvested at 48 h post-transfection and used to infect cells that were then selected with puromycin (8 µg/mL) for 7 days. Knockdown efficiency was determined by immunoblotting analysis.

### Co-IP assay

Cells were harvested and lysed in 1 mL of lysis buffer (PBS/1% NP-40, 5 mM EDTA, 5 mM EGTA) and clarified by centrifugation at 16,000 × *g* for 10 min at 4°C. Then, 900 µL aliquots were incubated with 40 µL of a 1:1 slurry of Sepharose conjugated with either IgG (17-0969-01, GE Healthcare) or an anti-FLAG mouse monoclonal antibody (A2220, Sigma-Aldrich) for 4 h at 4°C. The beads were washed three times with lysis buffer and eluted with SDS sample buffer by boiling for 10 min before immunoblotting analysis.

### ELISA

Concentrations of ILs were measured in the cell supernatants with ELISA kits from R&D Systems (IL-1β, PLB00B) and Advanced BioChemical (IL-18, ABCE-EL-P007) according to the manufacturers’ instructions.

### Caspase-1 activity assay

Caspase-1 activity was assessed with a Caspase-Glo 1 Inflammasome Assay kit (G9951, Promega) with cell lysates treated as indicated according to the manufacturer’s instructions.

### ATP assay

Cellular ATP was measured by the Enhanced ATP Assay Kit (S0027) according to the manufacturer’s instructions.

### NAD^+^/NADH assay

The ratio of NAD^+^/NADH was measured by NAD+/NADH Assay Kit with WST-8 (S0175) according to the manufacturer’s instructions.

### Mitochondrial membrane potential (Δψm) assay

Changes in mitochondrial membrane potential were detected by using a mitochondrial membrane potential assay kit with JC-1 (Beyotime) according to the manufacturer’s instructions. Briefly, the cells were seeded in 35 mM dishes at a density of 1 × 10^5^ cells per dish. After the indicated treatment, cells were incubated with JC-1 for 20 min. For imaging of JC-1 monomers, a Zeiss LSM 800 confocal microscope was set at 490 nm excitation and 530 nM emission wavelengths; for JC-1 aggregates, the wavelengths were set at 525 nm excitation and 590 nm emission.

### PLA assay

Cell fixation and permeabilization were performed as described in the immunofluorescence analysis. Duolink II *in situ* PLA (38534, Sigma-Aldrich) enables the detection, visualization, and quantification of protein interactions (˂40 nm) as an individual fluorescent dot by microscopy. The proximity ligations were performed according to the manufacturer’s instructions. Cells were mounted in Duolink II mounting medium containing DAPI (DUO82040, Sigma-Aldrich). Fluorescence was analyzed with a Zeiss inverted fluorescent microscope equipped with an ApoTome using the AxioVision program. Quantification of signals was done with the ImageJ software and expressed as a percentage of blobs per cell compared with the control. Experiments were performed at least three times, with a minimum of five fields taken per condition.

### Calcium assay

MARC-145 cells were loaded with membrane-permeable Fluo-3-AM (5 µM), a fluorescent Ca^2+^ indicator, for 10 min at 37°C in the dark. An excitation wavelength of 488 nm was provided by a Zeiss LSM 800 confocal microscope, and fluorescence signals were examined using a 515 nM pass emission filter. Background fluorescence was subtracted from all signals. Changes in [Ca^2+^] were determined using changes in fluorescence expressed as F/F0, where F0 is the control, diastolic fluorescence. The change in fluorescence intensity after drug treatment was normalized using the initial intensity.

### Statistical analysis

All data were analyzed using Prism 8 software (GraphPad) and a two-tailed Student’s *t*-test. *P* < 0.05 was considered statistically significant. Data are shown as the mean ± standard deviation from three independent experiments.

## SUPPLEMENTAL MATERIAL

Supplemental figures mbio.02651-23-s0001.docx Fig. S1 to S4.

Table S1 mbio.02651-23-s0002.docx Primers.

Table S2 mbio.02651-23-s0003.docx shRNAs.

ASM does not own the copyrights to Supplemental Material that may be linked to, or accessed through, an article. The authors have granted ASM a non-exclusive, world-wide license to publish the Supplemental Material files. Please contact the corresponding author directly for reuse.
