# Supplementary material for: The glycoprotein 5 of porcine reproductive and respiratory syndrome virus stimulates mitochondrial ROS to facilitate viral replication
Source: mBio. 2023 Dec 4;14(6):e02651-23. doi: 10.1128/mbio.02651-23 (PMC10746205; doi:10.1128/mbio.02651-23)
Supplement: Supplemental figures — Fig. S1 to S4. [file mbio.02651-23-s0001.docx]

**Supplemental Materials for**

**The glycoprotein 5 of porcine reproductive and respiratory syndrome virus stimulates mitochondrial ROS to facilitate viral replication**

Shuang Zhang, Lei Zeng, Bing-Qian Su, Guo-Yu Yang, Jiang Wang, Sheng-Li Ming, Bei-Bei Chu

**This file includes Figure S1 to S4.**


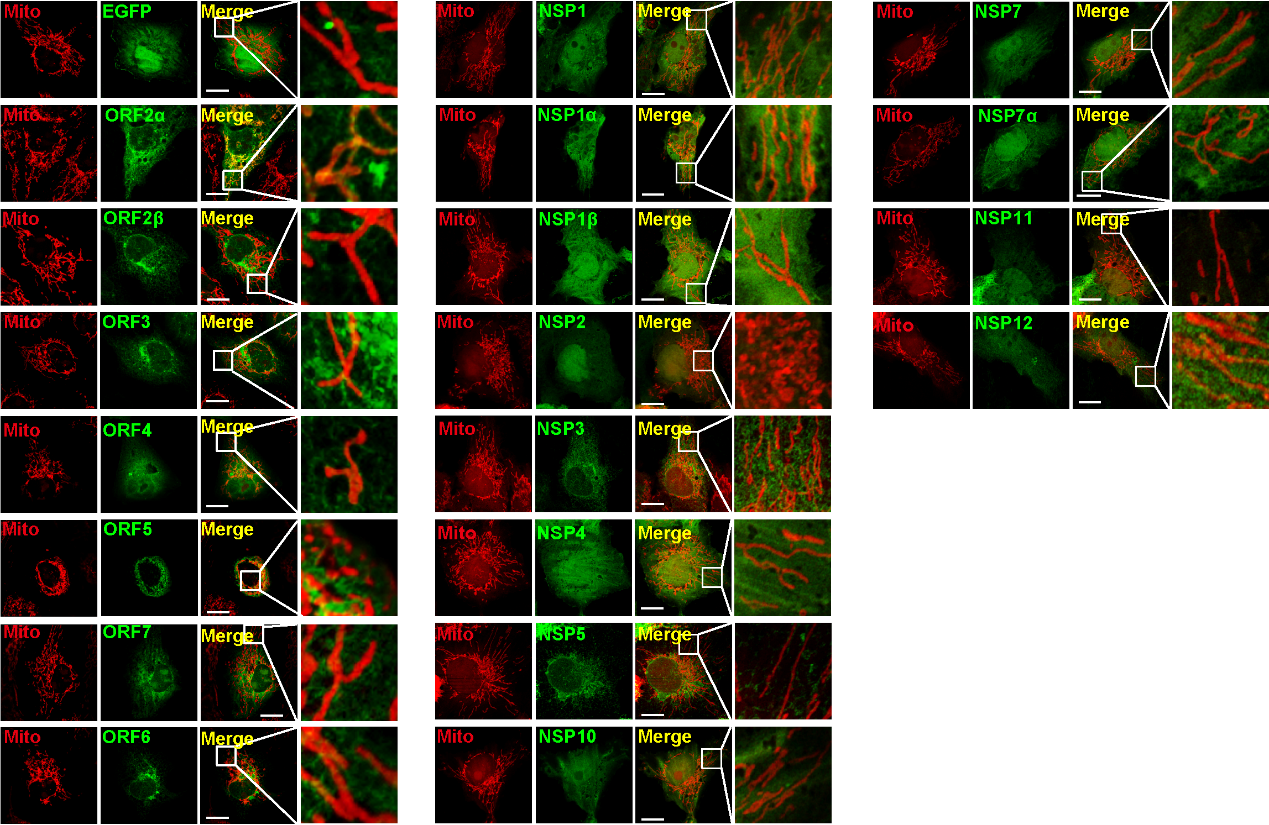


**Figure S1. Screening for PRRSV ORFs that alter mitochondria morphology.** MARC-145 cells were transfected with the indicated PRRSV ORFs for 24 h. The morphology of mitochondria (Tom20) was monitored by immunofluorescence analysis. Scale bar: 10 μm.


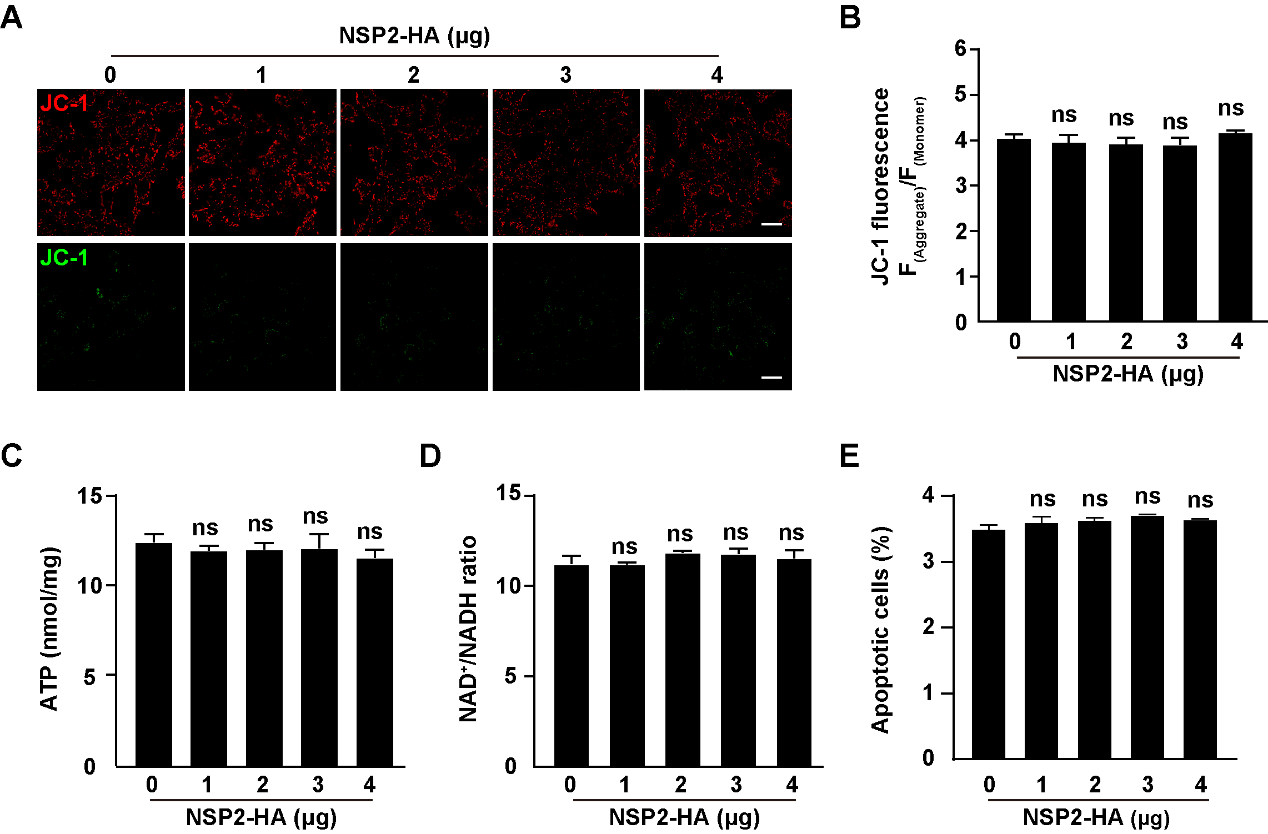


**Figure S2. PRRSV NSP2 has little impact on mitochondrial dysfunction.** (A) MARC-145 cells were transfected with NSP2-HA (0–4 μg) for 24 h. The mitochondrial membrane potential was analyzed by JC-1 staining. Scale bar: 10 μm. (B) Quantification of the ratio of aggregated and monomer JC-1 from (A). ns, no significance. (C) MARC-145 cells were transfected with NSP2-HA (0–4 μg) for 24 h. Cellular ATP was measured using an ATP assay kit. ns, no significance. ns, no significance. (D) MARC-145 cells were transfected with NSP2-HA (0–4 μg) for 24 h. The NAD^+^/NADH ratio was measured by the NAD^+^/NADH assay kit. ns, no significance. (E) MARC-145 cells were transfected with NSP2-HA (0–4 μg) for 48 h. Apoptosis assay was performed by Annexin V FITC and PI staining. ns, no significance.


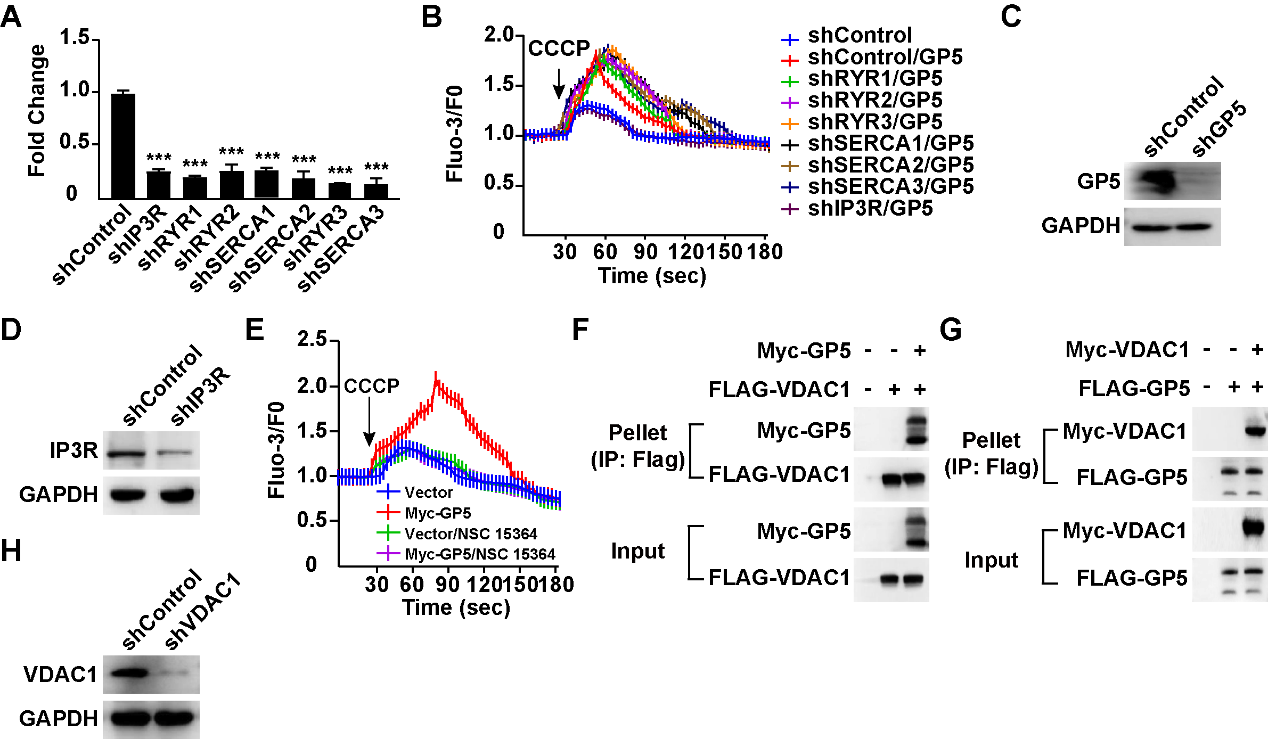


**Figure S3. Screening for ER Ca^2+^ channel that mediates ER Ca^2+^ release into mitochondria.** (A) The mRNA levels of IP3R, RYR1, RYR2, SERCA1, SERCA2, RYR3 and SERCA3 in their knockdown MARC-145 cells were analyzed by qRT-PCR analysis. ****P* < 0.001. (B) shControl, shIP3R, shRYR1, shRYR2, shSERCA1, shSERCA2, shRYR3 and shSERCA3 MARC-145 cells were transfected with GP5-EGFP (4 μg) as indicated for 24 h and then treated with CCCP (10 μM) to release mitochondrial Ca^2+^. (C) shControl and shGP5 MARC-145 cells were infected with PRRSV (MOI = 1) for 48 h. GP5 was analyzed by immunoblotting analysis. (D) IP3R was analyzed by immunoblotting analysis in shControl and shIP3R MARC-145 cells. (E) MARC-145 cells were transfected with vector or Myc-GP5 and treated with vehicle or NSC 15364 (50 μM) for 24 h. CCCP (10 μM) was used to release mitochondrial Ca^2+^. (F) MARC-145 cells were co-transfected with Myc-GP5 and FLAG-VDAC1 for 24 h. The interaction of Myc-GP5 with FLAG-VDAC1 was analyzed by CoIP analysis. (G) MARC-145 cells were co-transfected with Myc-VDAC1 and FLAG-GP5 for 24 h. The interaction of Myc-VDAC1 and FLAG-GP5 was analyzed by CoIP analysis. (H) VDAC1 was analyzed by immunoblotting analysis in shControl and shVDAC1 MARC-145 cells.


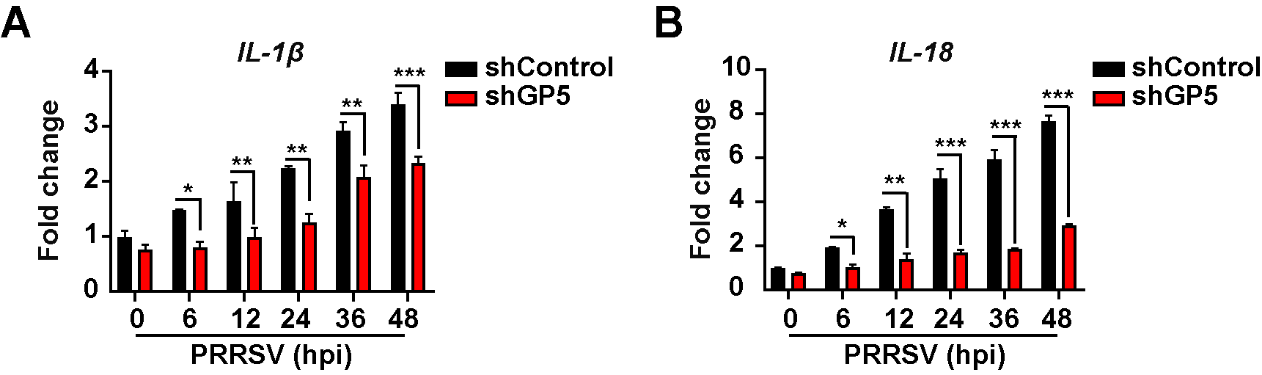


**Figure S4. Knockdown of GP5 inhibits PRRSV-activated NLRP3 inflammasome.**

(A and B) shControl and shGP5 iPAMs were infected with PRRSV (MOI = 1) for 0–48 h. The mRNA levels of IL-1β (C) and IL-18 (D) were analyzed by qRT-PCR analysis. ^*^*P* < 0.05, ^**^*P* < 0.01, ^***^*P* < 0.001.
