# Supplementary material for: The glycoprotein 5 of porcine reproductive and respiratory syndrome virus stimulates mitochondrial ROS to facilitate viral replication
Source: mBio. 2023 Dec 4;14(6):e02651-23. doi: 10.1128/mbio.02651-23 (PMC10746205; doi:10.1128/mbio.02651-23)
Supplement: Table S1 — Primers. [file mbio.02651-23-s0002.docx]

**Table S1. List of primers used in this study.**

| **Genes** | **Forward (**5′-3′**)** | **Reverse (**5′-3′**)** |
| --- | --- | --- |
| SOD-2 | GTGGAGAACCCAAAGGGGAG | GCCTGTTGTTCCTTGCAGTG |
| ACTB | CGTGGACATCCGTAAAGAC | GGAAGGTGGACAGCGAGGC |
| HO-1 | ACTCCCTGGAGATGACTCCC | AGTCTTGCGCTTTGTTGCTG |
| MFN1 | TCCGCCTTTAACTTCTCGGG | ACAGTTTCTGCCATTATGTGTCTC |
| MFF | TGACCACGAACACTCTTCCG | GTGCTGGATTGAGAGCCACT |
| OPA1 | CTGAGTACGGGTGCCTGTC | CGTTCAGTAGCAAGGGCACA |
| PINK1 | CCTGGAGTGTGAAACGCTCT | CTCCCACCCTCACCATTCAC |
| ATF4 | CAGCATAGCCCGTCTACCAG | TCTCCAGGAGGGTCGTAAGG |
| DRP1 | ATGGCTGCTGACATTTCGGA | TGTTGGTAATGAACAGACTCCACA |
| PARKIN | GCATGCACATGAAGTGTCCG | GACACTGTGTATGCTCCCCC |
| IL-18 | GAGAAGTGTCCCAGGACATGA | GTGAGCCACTGCGCCC |
| IL-1β | TCCAGGGACAGGATCTGGAG | AACACGCAGGACAGGTACAG |
| VDAC1 | TGAGCAGAAGATGGCTGTGC | GGCTGAGCCTGAGCTTGTAA |
| IP3R | ACTGGCTGGTGGCTTTCATT | ACCTGGTAATCAAACCGATTGT |
| RYR1 | TGGGATTCCTGCACGTGTTC | CTGCAAAGGGGTGAGGGAAA |
| RYR2 | TGAAAGCATCAAACGCAGCA | ACTCGGCTCCACCTGATAGT |
| RYR3 | TTTAGGGGACATCCTCCGGT | CTCGGTGGAGAGCGCAAC |
| SERCA1 | CCATCTCTTTGAGCCCGTGT | ACAGACACATGACAGGGGTG |
| SERCA2 | CGAACCCTTGCCACTCATCT | CCAGTATTGCAGGTTCCAGGT |
| SERCA3 | ACCACGTGGACGAAAAGAGC | TCGGGCCTATCATCTCTCCG |
