# Supplementary material for: The glycoprotein 5 of porcine reproductive and respiratory syndrome virus stimulates mitochondrial ROS to facilitate viral replication
Source: mBio. 2023 Dec 4;14(6):e02651-23. doi: 10.1128/mbio.02651-23 (PMC10746205; doi:10.1128/mbio.02651-23)
Supplement: Table S2 — shRNAs. [file mbio.02651-23-s0003.docx]

**Table S2. List of shRNAs used in this study.**

| **Genes** | **Sequence (**5′-3′**)** |
| --- | --- |
| shControl | GCCACAACGTCTATATCATGG |
| shGP5 | GAGCTGAATGGCACAGATTGG |
| shIP3R | GACTTTGAGGAAGAATGCCTGGAGT |
| shRYR1 | CAGCCTTGCCAATGTCTCATGAACA |
| shRYR2 | GAGTTTGATCCTGACTTCCTGCAGT |
| shRYR3 | GGATGAAGAAGATGATGAAGA |
| shSERCA1 | GAGGATCCAGAAGATGAAAGA |
| shSERCA2 | GCAGGACATCAATGAGCAAGA |
| shSERCA3 | GCATCTTCCTCACGGCAATTC |
| shVDAC1 | AAGTGACGGGCAGTCTGGAAACCAA |
